# Enhancer RNA Transcriptome‐Wide Association Study Reveals a Distinctive Class of Pan‐Cancer Susceptibility eRNAs

**DOI:** 10.1002/advs.202411974

**Published:** 2025-02-14

**Authors:** Wenyan Chen, Zeyang Wang, Yinuo Wang, Jianxiang Lin, Shuxin Chen, Hui Chen, Xuelian Ma, Xudong Zou, Xing Li, Yangmei Qin, Kewei Xiong, Xixian Ma, Qi Liao, Yunbo Qiao, Lei Li

**Affiliations:** ^1^ Institute of Systems and Physical Biology Shenzhen Bay Laboratory Shenzhen 518055 China; ^2^ Ninth People's Hospital Shanghai Jiao Tong University School of Medicine Shanghai 200125 China; ^3^ Shanghai Institute of Precision Medicine Shanghai 200125 China; ^4^ School of Public Health Health Science Center Ningbo University Ningbo 315211 China

**Keywords:** enhancer RNA, genome‐wide association study, noncoding variants, pan‐cancer, transcriptome‐wide association study

## Abstract

Many cancer risk variants are located within enhancer regions and lack sufficient molecular interpretation. Here, we constructed the first comprehensive atlas of enhancer RNA (eRNA)‐mediated genetic effects from 28 033 RNA sequencing samples across 11 606 individuals, identifying 21 073 eRNA quantitative trait loci (eRNA‐QTLs) significantly associated with eRNA expression. Mechanistically, eRNA‐QTLs frequently altered binding motifs of transcription factors. In addition, 28.48% of cancer risk variants are strongly colocalized with eRNA‐QTLs. A pan‐cancer eRNA‐based transcriptome‐wide association study is conducted across 23 major cancer types, identifying 626 significant cancer susceptibility eRNAs predicted to modulate cancer risk via eRNA, from which 54.90% of the eRNA target genes are overlooked by traditional gene expression studies, and most are essential for cancer cell proliferation. As proof of principle validation, the enhancer functionality of two newly identified susceptibility eRNAs, *CCND1e* and *SNAPC1e*, is confirmed through CRISPR inhibition and shRNA‐mediated knockdown, resulting in a marked decrease in the expression of their respective target genes, consequently suppressing the proliferation of prostate cancer cells. The study underscores the essential role of eRNA in unveiling new cancer susceptibility genes and establishes a strong framework for enhancing our understanding of human cancer etiology.

## Graphical abstract

Enhancer RNA (eRNA) can play a key role in cancer initiation and progression. Here, the authors conducts a comprehensive pan‐cancer eRNA‐based TWAS analysis and discovered a distinct class of eRNA‐mediated cancer susceptibility genes across 23 cancer types.

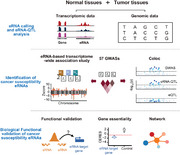

## 1 Introduction

Genome‐wide association studies (GWASs) have identified numerous single‐nucleotide polymorphisms (SNPs) associated with complex human traits and disorders.^[^ ^1^ ^]^ However, most of these SNPs are located in noncoding regions of the genome,^[^ ^2^ ^]^ particularly in enhancer regions.^[^ ^3^, ^4^, ^5^ ^]^ Expression quantitative trait loci (eQTLs) often serve as a crucial link between GWAS SNPs and disease phenotypes, providing valuable insights into how genetic variants influence the regulation of nearby gene transcription and further contribute to the disease risk. Despite these advances, the functional roles of many disease‐associated variants, especially those that reside in noncoding regulatory regions, remain largely unknown.

Enhancer RNAs (eRNAs) are a class of noncoding RNAs transcribed from active enhancer regions.^[^ ^6^, ^7^, ^8^ ^]^ Their transcription is closely associated with RNA polymerase II (RNAPII) activity and epigenetic modifications, such as H3K27ac and H3K4me1 histone marks.^[^ ^9^ ^]^ eRNAs can independently regulate the expression of nearby genes. For instance, eRNAs transcribed from p53‐bound enhancers play a pivotal role in DNA damage response by enhancing the transcription of genes involved in cell cycle arrest and apoptosis.^[^ ^10^, ^11^ ^]^ Moreover, eRNAs contribute significantly to disease progression,^[^ ^12^ ^]^ particularly in cancer.^[^ ^13^, ^14^ ^]^ One example is *CCAT1e*, an eRNA that interacts with the transcription factor *TCF7L2* to activate the Wnt signaling pathway, promoting colon cancer cell proliferation and invasion.^[^ ^13^, ^15^, ^16^ ^]^ Similarly, *KLK3e* and *PSAe*, eRNAs regulated by the androgen receptor, influence androgen‐induced gene activation and contribute to prostate cancer development.^[^ ^17^, ^18^ ^]^ Despite these individual examples, the prevalence and magnitude of eRNAs influencing cancer susceptibility remain largely unknown.

Several specialized experimental methods, such as nuclear run‐on followed by cap‐selection assay (GRO/PRO‐cap) and self‐transcribing active regulatory region sequencing (STARR‐seq), can detect actively transcribed eRNAs.^[^ ^19^ ^]^ However, these techniques have not been widely adopted in large‐scale population studies. In contrast, RNA sequencing (RNA‐seq) has been extensively used in many genomic projects, such as Genotype‐Tissue Expression (GTEx),^[^ ^20^ ^]^ and recent studies have demonstrated the ability of RNA‐seq to accurately identify eRNAs.^[^ ^9^, ^13^, ^14^ ^]^ Moreover, eRNA‐associated genetic changes have been linked to the development of multiple diseases, including schizophrenia,^[^ ^21^ ^]^ and in human brains.^[^ ^22^ ^]^ However, the broad implication of genetic determinants impacting eRNA in various human tissues and their association with human cancers has not been systematically examined.

In this study, we performed the first large‐scale, systematic analysis assessing eRNA‐mediated genetic effects on 49 human normal tissues and 31 tumor tissues by analyzing 28 033 RNA‐seq samples from 11 606 individuals. Our analysis revealed that eRNA‐QTLs frequently disrupt transcription factor binding motifs, leading to altered changes in eRNA expression. We validated this using CRISPR‐based base editing experiments. Additionally, we conducted transcriptome‐wide association studies based on eRNA expression (eRNA‐TWAS), facilitating the functional characterization of cancer risk loci. We also demonstrated the enhancer activity of two newly identified susceptibility eRNAs (*CCND1e*, *SNAPC1e*) through CRISPR inhibition, which suppressed the proliferation of prostate cancer cells by regulating their target genes. Lastly, we developed a comprehensive online resource called the eRNA‐QTL atlas (https://bioinfo.szbl.ac.cn/eRNA‐QTL‐atlas/), providing researchers with access to the extensive data generated in this study. Overall, our findings substantially advance the understanding of the eRNA‐mediated genetic effects and their contribution to cancer risk.

## 2 Results

### 2.1 Atlas of eRNA‐Mediated Genetic Effects

**Figure 1:**
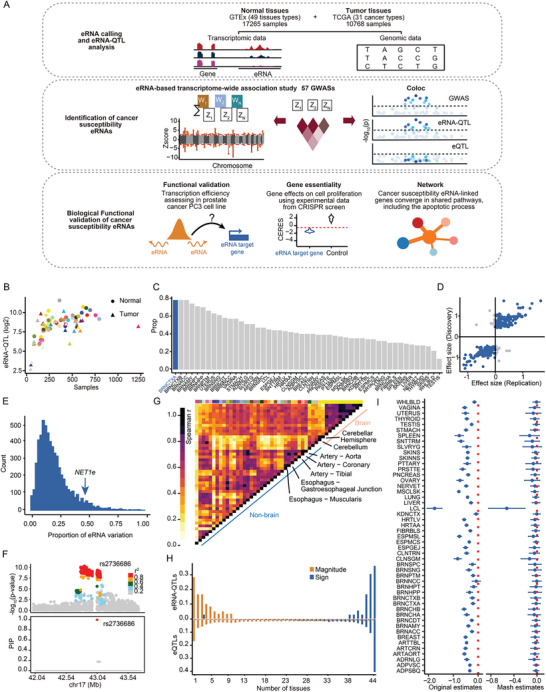
QTL mapping on eRNA transcriptome profiles across normal and tumor tissues. (A) Overview of the study and data. We integrated RNA‐seq and genotype data from GTEx and TCGA to develop a reference panel for eRNA‐QTL analysis and eRNA‐TWAS modeling. We then employed eRNA‐TWAS analysis to identify cancer susceptibility eRNA‐link genes using cancer GWAS summary statistics and eRNA‐TWAS models. The biological functions of these cancer susceptibility eRNA‐link genes were further validated by data analysis and experimental approach. (B) The number of identified eRNA‐QTLs in normal and tumor tissues, along with the corresponding number of tissue samples. The numbers of eRNA‐QTLs are transformed using log2 scale. Circles represent normal tissue, and triangles represent tumor tissue. The color coding is detailed in Table S1 (Supporting Information). (C) Replication in the Independent Dataset (PsychENCODE). The PsychENCODE dataset comprises samples from the brain cortex. The QTL mapping reveals that the overlap of significant eRNAs identified in the replication cohort (PsychENCODE) with those identified in the discovery cohort (GTEx) for the same tissue type is the highest. The overlap rates for significant eRNAs identified in other tissues are comparatively lower. (D) A scatter plot of effect sizes (beta coefficients) for eRNA‐QTLs identified in both the discovery cohort (GTEx) and the replication cohort (PsychENCODE). (E) Average fraction of eRNA variations that could be explained by eRNA‐QTLs. The y‐axis represents the proportion of eRNAs across all human normal (GTEx) tissues and tumor (TCGA) types studied. (F) *BRCA1* eRNA (*BRCA1e*), located upstream of the *BRCA1* gene, contains an independent eRNA‐QTL rs2736686. (Top panel) Genetic variants are depicted as points color‐coded according to their linkage disequilibrium (LD) with the candidate variant rs6866783 (red, ≥ 0.8; orange, 0.6‐0.8; green, 0.4‐0.6; light blue, 0.2‐0.4; grey, < 0.2). LD data is sourced from the 1,000 Genomes Project (Phase 3). The bottom panel consolidates the outcomes of applying SusieR to the eRNA‐QTL summary data, where SusieR identifies a credible set denoted by red circles, encompassing the putative causal SNP rs2736686. PIP refers to posterior inclusion probability. A higher PIP indicates a greater likelihood that the SNP is a causal SNP. (G) Pairwise eRNA‐QTL sharing by magnitude among tissues. eRNA‐QTL sharing patterns were assessed pairwise across various tissues by examining the Spearman correlation between mashR effect sizes for each tissue pair. The results are displayed in matrix format, with each cell representing the correlation value for a particular tissue pair. To identify shared eRNA‐QTLs between two tissues, the top eRNA‐QTLs that attained significance (local false sign rate < 0.05) in at least one of the two tissues were selected. The proportion of shared eRNA‐QTLs was then plotted, whereby only those with effect estimates of the same sign and within a factor of 2 in size were included. The hierarchical clustering algorithm was utilized to arrange the tissues based on their similarity in eRNA‐QTL sharing patterns. The color and shape of each dot refers to the tissue recorded in the GTEx dataset (Table S1, Supporting Information). (H) Proportion of tissues sharing lead eRNA‐QTLs/eQTLs across all 49 examined tissues. (I) The original estimates and mash estimates of eRNA‐QTL effect size for *CCDC32e*. The lines depict the median and the shadings of the 95% confidence intervals (CIs).

To systematically detect eRNAs in human primary tissues, we annotated and quantified eRNA expression using RNA‐seq data following previously described methods.^[^ ^9^ ^]^ We integrated datasets from ENCODE, FANTOM, and Roadmap Epigenomics to annotate enhancers, considering only those present in at least two datasets. To avoid overlapping with known transcripts, we retained only intergenic eRNAs that did not overlap with existing annotations, such as protein‐coding RNAs and non‐coding RNAs (ncRNAs). We detected 12 509 eRNAs in 17 265 RNA‐seq samples from 49 normal human tissues and 9111 eRNAs in 10 768 RNA‐seq samples from 31 tumor tissues (**Figure** **1**A). We further performed clustering analysis across all samples to assess whether eRNA expression profiles can differentiate human tissues and organs. This revealed distinct patterns of eRNA expression, with eRNA from the same organ tended to cluster together, clearly distinguishing them from other tissues, as exemplified by notably distinct eRNA expression profiles between the artery and heart (Figure S1A, Supporting Information). Interestingly, we observed that prostate tissue formed a distinct cluster based solely on eRNA expression profiles, whereas it did not segregate distinctly based on gene expression profiles alone (Figure S1A, Supporting Information). The distinct clustering of prostate tissue based on eRNA expression profiles indicates that eRNAs could have tissue‐specific expression patterns or functions that are not captured by traditional gene expression analysis.

Next, we investigated the influence of genetic variations on eRNA expression. Following the normalization of eRNA expression, adjusted for known covariates, and determined the optimal number of principal components derived from eRNA expression (see Experimental Section; Figure S1B,C, Supporting Information), we then used a linear regression model implemented in QTLtools version 1.2^[^ ^23^ ^]^ to identify common genetic variants associated with eRNA expression in each tissue or cancer type. Applying a false discovery rate (FDR) threshold of 0.05, we identified 21 073 eRNA‐QTLs (i.e., genetic variants associated with eRNA expression) associated with 89.75% of annotated eRNAs. This includes 11 757 eRNA‐QTLs from normal tissues and 9,316 from tumor tissues (Figure 1B). The genomic inflation factors for eRNA‐QTLs ranged from 0.95 to 1.17 across tissues, indicating well‐controlled population stratification (Figure S1D, Supporting Information E). The number of eRNA‐QTLs varied widely among the tissues, ranging from 57 in the brain substantia nigra to 3022 in the testis (Table S1, Supporting Information). Notably, tissues with large sample sizes tended to have more eRNA‐QTLs (Rho = 0.99, *P* < 0.001; Figure 1B). To validate these findings, we applied our pipeline to another independent dataset^[^ ^24^ ^]^ and observed a high replication rate of eRNA‐QTLs (π_1_ statistic = 0.93). Specifically, 78% of significant eRNAs in the brain cortex were replicated in the validation brain cohort, compared to 38% in other tissues (Figure 1C). Furthermore, the effect sizes of eRNA‐QTLs were consistent between cohorts (Figure 1D), with no significant difference (*P* = 0.38), confirming robust replication. In addition, we also selected six representative tissues for eRNA‐QTL mapping using a linear mixed model implemented in lme4qtl.^[^ ^25^ ^]^ The effect sizes of eRNA‐QTLs identified by both models were well consistent across all tissues (Figure S2A–F, Supporting Information). Additionally, previously known eRNA‐QTLs were successfully recovered, such as rs72700813 (Figure S3A, Supporting Information), which modulates *GOLPH3L* eRNA (*GOLPH3Le*) expression^[^ ^21^ ^]^ and is related to *GOLPH3L*, a prognostic biomarker for epithelial ovarian cancer.^[^ ^26^ ^]^

We then estimated the heritability of eRNAs using genome‐wide complex trait analysis (GCTA) with the GREML method.^[^ ^27^ ^]^ Our analysis revealed that eRNA‐QTLs collectively explained an average of 24.4% of eRNA variation in normal tissues and 20.97% in tumor tissues (Figure S3B, Supporting Information). For example, *NET1e*, an oncogene‐associated eRNA in breast, prostate, and liver cancer,^[^ ^13^ ^]^ exhibited a heritability estimate of 0.46 (*P* < 2.2 × 10^−16^; Figure 1E). Conditional stepwise regression revealed that similar to eQTL,^[^ ^20^ ^]^ 77.25% of eRNAs in normal tissues and 76.03% in tumor tissues contained one independent eRNA‐QTL (Figure S3C, Supporting Information). For example, the *BRCA1*‐associated eRNA (*BRCA1e*) contained only one independent eRNA‐QTL, rs2736686 (posterior inclusion probability (PP) = 1; Figure 1F), highlighting the regulatory influence of individual genetic variants on eRNA expression. To further explore the sharing patterns of eRNA‐QTLs across tissues, we applied the multiple adaptive shrinkage (Mash) model^[^ ^28^ ^]^ to calculate pairwise eRNA‐QTL sharing. eRNA‐QTLs were clustered into two distinct groups: brain and non‐brain tissues (Figure 1G). We also observed that a majority of eRNA‐QTLs exhibited tissue‐specificity, in contrast to eQTLs, which are more frequently shared across different tissues (Figure 1H; Figure S3D, Supporting Information). For instance, the eRNA‐QTL effects associated with the *CCDC32e* (Figure 1I) exhibited an exclusively strong effect size in lymphoblastoid cells (LCL). Taken together, we constructed a comprehensive atlas of eRNA‐mediated genetic effects across 49 human normal tissues and 31 tumor tissues, highlighting the discriminative potential of eRNA‐QTLs among various biological tissues.

### 2.2 Distinct Regulatory Role of eRNA‐QTLs Compared to eQTLs

**Figure 2:**
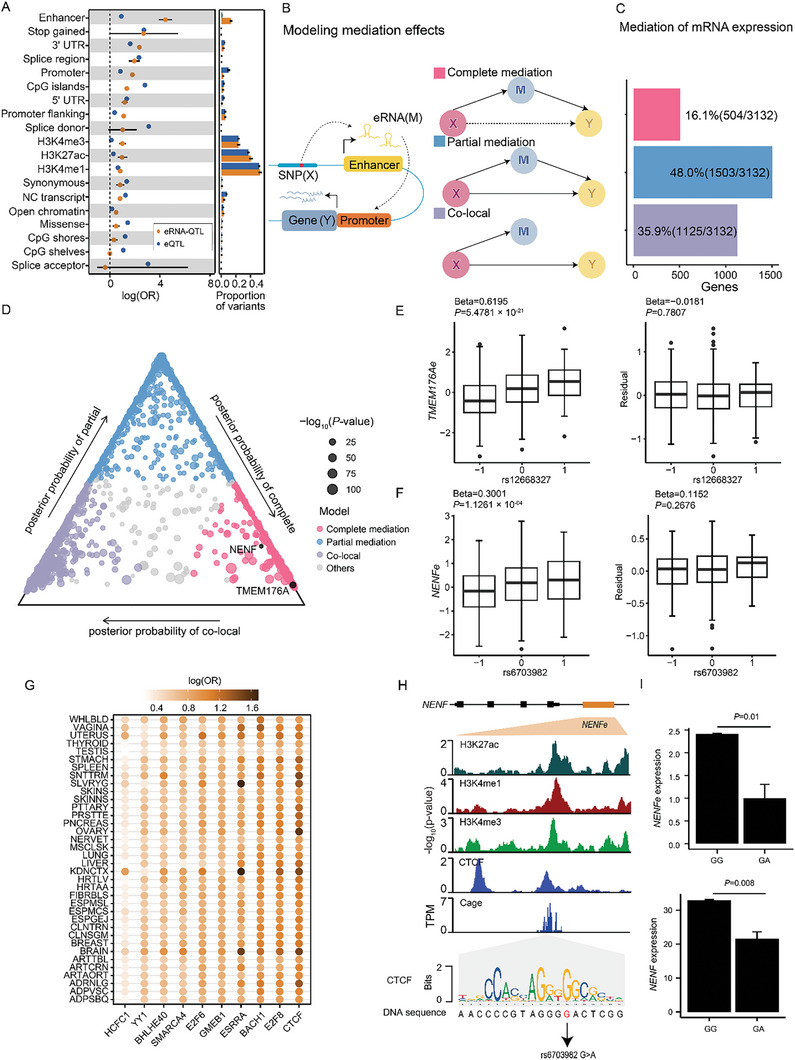
Functional annotation of eRNA‐QTLs. (A) Enrichment of eRNA‐QTLs and eQTLs for different genome annotations. Each dot in the figure represents the log‐transformed odds ratio (OR), and the lines indicate the corresponding 95% confidence interval. Enrichment was assessed using all SNPs through torus based on the eRNA‐QTL results obtained using QTL tools and the eQTL results obtained from the GTEx Portal. The peak files of histone modification markers were downloaded from the Roadmap Epigenomics Project. The bar plot on the right panel illustrates the proportion of lead eQTLs and lead eRNA‐QTLs in various annotations. The error bars represent the 95% confidence interval of the mean. (B) Relationships modeled in the mediation testing. In the complete mediation model and the partial mediation model, the effect of a SNP on a phenotype Y (eQTL) is completely or partially explained by the effect of the same SNP on a phenotype M (eRNA‐QTL). The co‐local model represents cases where the SNP independently affects without mediation between M and Y. (C) Proportion of 3,144 genes with significant eQTL mediated by eRNA‐QTLs according to each model. (D) The posterior probabilities of different models for each X‐M‐Y triplets in the test. The point size represented the ‐log10 nominal P‐value of the corresponding eRNA‐QTL. (E‐F) Examples of complete mediations for eQTL for the gene *TMEM176A* (E) and *NENF* (F) via an eRNA‐QTL, respectively. The box plots show the associations of the SNP with the eRNA expresssion and the residual expression of genes after regressing the effects of the eRNA expression. The box plots show the median in the central line, the box spans the first to the third quartiles and the whiskers extend 1.5 times the IQR from the box. Slopes (beta) and nominal P‐values are shown for each association (linear regression model). (G) eRNA‐QTLs exhibited significant enrichment within the top ten TFBSs, which were associated with the highest number of tissues showing substantial enrichment across diverse tissue types. The depth of color represents the maximum‐likelihood estimated log(OR), with darker colors indicating a higher value. In the case of eRNA‐QTLs, darker colors indicate a higher enrichment in TFBSs, while lighter colors indicate lower enrichment. The enrichment results for all transcription factor binding sites (TFBSs) can be found in Table S2 (Supporting Information). (H) The genome browser displays histone modification data, CAGE data, and CTCF binding site data within the *NENF* eRNA (*NENFe*) locus. For the histone modification data and the CTCF binding site data, the values on the y‐axis correspond to signal *p*‐value. For the CAGE data, the values on the y‐axis correspond to the maximum normalized TPM (Transcripts Per Million). Histone modification data were downloaded from the Roadmap Epigenomics Project, CAGE data were obtained from FANTOM5, and CTCF binding site data were acquired from Cistrome. A DNA logo is presented representing the CTCF‐binding motif based on previously reported consensus CTCF binding sites.^[^ ^64^ ^]^ The height of each letter in the logo indicates the relative frequency of occurrence of the corresponding nucleotide at that specific position. (I) The effects of different genotypes at rs6703982 on the expression of *NENF* eRNA (*NENFe*) and its target gene *NENF*. The expression of *NENFe* was normalized using RPM, while the expression of *NENF* was normalized using TPM. The A allele of rs6703982 was observed to downregulate the expression of *NENFe* and its target gene *NENF*.

To characterize the relationship between eRNA‐QTLs and other molecular QTLs such as eQTLs, we conducted functional annotation enrichment of lead eRNA‐QTLs and eQTLs using torus^[^ ^29^ ^]^ with a FDR threshold of 5%. We found that eRNA‐QTLs were significantly enriched in enhancer (4.44‐fold enrichment compared with background SNPs; two‐sided Fisher's exact test, adjusted *P* = 8.43 × 10^−59^) and in promoter regions (1.8‐fold enrichment compared with matched background SNPs; two‐sided Fisher's exact test, adjusted *P* = 2.43 × 10^−114^) (**Figure** **2**A). This observation is similar with our positional distribution analysis, most lead eRNA‐QTLs were enriched within eRNA regions (Figure S3E, Supporting Information).

eRNA can interact with chromatin to modify chromatin accessibility and impact histone marks in the promoter of the target gene, especially the acetylation and methylation of H3K27, which in turn affects the expression of the target gene.^[^ ^30^, ^31^ ^]^ To investigate to what extent eRNA may act as an intermediate for the SNP effects on gene expression, we conducted the mediation analysis using bmediatR based on the Bayesian model^[^ ^32^ ^]^ (Figure 2B). Briefly, three causal relationship models were tested: i) Complete mediation model (top), wherein eRNA (M) completely determines the effects of SNPs on the gene expression (Y); ii) Partial mediation model (middle), wherein eRNA only partially dictates the effects of SNPs on the gene expression; and iii) Co‐local model (bottom), wherein the effects of SNPs on both eRNA and gene expression are co‐localized but independent. Our analysis identified 70 927 SNP‐eRNA‐gene triplets, comprising 3132 genes linked to 22 403 genetic variants and 2881 eRNAs (Figure S3F, Supporting Information), and 78.27% of these triplets showed allelic effects in the same direction on both eRNA and gene expression (Figure S3G, Supporting Information). Remarkably, 64.1% of the eRNA‐QTLs were mediated by eRNAs, either completely or partially, with 16.1% of eRNA‐QTLs showing complete mediation (Figure 2C). Notable examples of complete mediation include the eRNA‐QTL‐mediated effects on *TMEM176A* and *NENF* genes (Figure 2D–F). For instance, SNP rs12668327 was found to influence both *TMEM176A* gene expression (*P* = 1.71 × 10^−6^) and the expression of *TMEM176A* eRNA (*TMEM176A*e) (*P* = 5.48 × 10^−21^). The variation of *TMEM176A* gene expression was found to be completely mediated through *TMEM176A*e (posterior probability of complete/partial: 0.91/0.081) (Figure 2D,E). After adjusting for eRNA effects of *TMEM176A*, the association of the rs12668327 with *TMEM176A* expression became insignificant (*P* = 0.78) (Figure 2E). *TMEM176A* is often associated with loss of heterozygosity in human cancers.^[^ ^33^ ^]^ Similarly, the variant rs6703982 was significantly associated with the expression of *NENF* eRNA (*NENFe*) (*P* = 1.13 × 10^−4^; Figure 2F). Furthermore, the variation of *NENF* gene expression was found to be completely mediated through *NENF*e (posterior probability of complete/partial: 0.571/0.186, Figure 2D). As before, the association of the rs6703982 with *NENF* expression became insignificant (*P* = 0.2676) after adjusting for eRNA effects of *NENF* (Figure 2F). *NENF* was shown to activate the ERK1/2 and AKT pathways in both mouse neurons and MCF‐7 breast cancer cells.^[^ ^34^ ^]^ Depletion of *NENF* reduced the tumorigenic properties of various cancer cells, including liver, bladder, and breast cancer.^[^ ^35^ ^]^ Taken together, our findings highlight the causal role of eRNA‐QTLs in regulating gene expression.

### 2.3 Alterations of Transcription Factor Binding Sites are Associated with eRNA Expression

To further elucidate the mechanisms through which these eRNA‐QTLs exert their effects, we hypothesized that some eRNA‐QTLs may disrupt transcription factor binding sites (TFBSs), thereby altering eRNA expression. To test this hypothesis, we analyzed ChIP‐seq data for 194 TFs from The Encyclopedia of DNA Elements (ENCODE) project.^[^ ^36^ ^]^ We examined whether eRNA‐QTLs were significantly enriched within the ChIP binding peaks of each TF compared with randomly shuffled sequence datasets. Our analysis identified 104 TFs that preferentially bound to regions containing eRNA‐QTLs in normal tissues and 83 TFs in tumor tissues (Table S2, Supporting Information). These included *CTCF*, *SMARCA4*, and *YY1*, which are known to regulate eRNA (Figure 2G). For example, SNP rs17776622, located within the core binding motif of the transcription factor *YY1*, was identified as the causal eRNA‐QTL of the *SOX7* eRNA (*SOX7e*) (Posterior Probability = 0.98, Figure S3H, Supporting Information). Moreover, *SOX7e* exhibited tight co‐regulation with the *SOX7* gene (Figure S3I, Supporting Information), suggesting that rs17776622 could impact *SOX7* gene expression by altering *YY1* binding motifs. Pathway enrichment analysis showed that these TFs are enriched in several important biological processes, including transcription activator activity (Fisher's exact test, adjusted *P* = 9.80**×**10^−7^) and polymerase II transcription activator activity (Fisher's exact test, adjusted *P* = 1.88**×**10^−6^) (Figure S3J, v). Interestingly, we observed CTCF with binding sites selectively bound to eRNA‐QTLs‐containing regions across most tissues (adjusted *P* < 0.05; Figure 2G). To further validate our hypothesis, we performed CRISPR‐based base editing experiments to mimic the alternative allele of eRNA‐QTL rs6703982, which is located within a *CTCF* binding motif region (Figure 2H). Using HEK293T cells, we successfully generated two clones with heterozygous G/A mutations (Figure S3K, Supporting Information). Consistent with our prediction, introducing the alternative allele at rs6703982 resulted in decreased *NENFe* expression and its associated target gene *NENF* (Figure 2I). Thus, our findings suggest that altered eRNA expression is elicited by eRNA‐QTLs that disrupt the binding motifs of TFs.

### 2.4 Most Cancer‐Co‐Localizing Cancer Loci Were Exclusively Detected by eRNA‐QTLs

**Figure 3:**
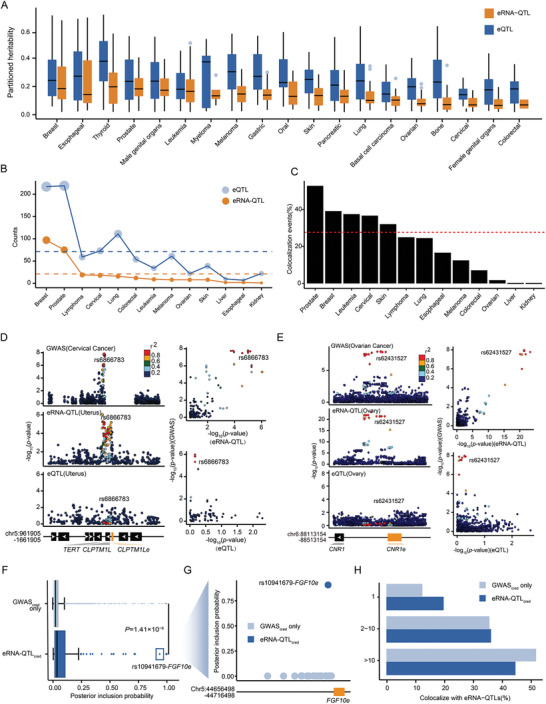
Contribution of eRNA‐QTLs to cancer heritability. (A) A significant contribution to the heritability of cancers by eRNA‐QTL/eQTL. Cancer types were grouped on the x‐axis. For each cancer type, the yellow and blue colors indicate the contribution of eRNA‐QTLs and eQTLs to the heritability of cancers, respectively. (B) Number of cancer risk loci co‐localizing with eRNA‐QTL and eQTL. Cancer types are grouped on the x‐axis. For each cancer type, yellow and blue colors indicate that the colocalization events could be explained by eRNA‐QTL or eQTL, respectively. (C) Proportion of cancer risk loci co‐localized with eRNA‐QTLs. The red dashed line represents the average proportion of co‐localization with eRNA‐QTLs across all cancer risk loci. (D) Independent eRNA‐QTLs depict variants with regulation on the eRNA level but not the mRNA displayed by the significant SNP‐eRNA pair rs6866783‐*CLPTM1Le*. Variants are represented by points colored relative to LD with the candidate variant rs6866783 (red, ≥ 0.8; orange, 0.6–0.8; green, 0.4–0.6; light blue, 0.2–0.4; dark blue, < 0.2). LD data from 1,000 Genomes (phase 3). (E) Independent eRNA‐QTLs depict variants with regulation on the eRNA level but not the mRNA level displayed by the significant SNP‐eRNA pair rs62431527‐*CNR1e*. (F) Distribution of PIP for all fine‐mapped GWAS variants (95% credible set in GWAS, GWAS_cred_) that were also fine‐mapped eRNA‐QTL variants (eRNA‐QTL_cred_) vs. GWAS_cred_ variants only. Mann‐Whitney *P‐*value is shown. (G) Elevated causal probability of cancer‐credible variants associated with eRNA‐QTLs compared to non‐eRNA‐QTLs variants, highlighted by the example of rs10941679 in the breast cancer risk locus 5p12. (H) Integration of cancer‐credible GWAS variants with credible sets from colocalizing eRNA‐QTLs increased fine‐mapping resolution. The bar plot shows the proportion of independent loci identified as candidate causal variants before and after restricting for QTL variants. The credible set sizes are binned into three groups (1, 2–10, and > 10).

To assess the prevalence of disease‐associated variants within eRNA region, we analyzed the genomic distribution of disease‐associated fine‐mapped variants (95% credible sets) from CAUSALdb.^[^ ^37^ ^]^ We observed that 70.47% of cancer‐associated variants overlapped with eRNAs, while only 44.68% for non‐cancer‐associated variants (Figure S4A, Supporting Information), indicating that cancer‐associated variants are significantly enriched within eRNA transcribing regions compared to variants not associated with cancer (Figure S4B, Supporting Information). For example, the prostate cancer risk SNP rs55714314 (*P* = 4.86**×**10^−12^) was identified in the eRNA region of the *GATA2* gene (Figure S4C, Supporting Information). Remarkably, it exhibited the highest posterior probability of being a causal SNP of prostate cancer (Figure S4C, Supporting Information). To further evaluate whether eRNA‐QTLs could be used to interpret cancer traits, particularly those located within regulatory regions, we compiled and curated 57 GWAS summary statistics covering 23 cancer types from the literature (Table S3, Supporting Information). Utilizing GARFIELD (v2),^[^ ^38^ ^]^ we analyzed the enrichment of eRNA‐QTLs within the cancer risk loci. We identified that eRNA‐QTLs were enriched within 25.69% of tissue‐trait pairs (i.e., pairs of 49 tissues and 57 GWAS traits) (see Methods; Figure S4D, Supporting Information). Moreover, eRNA‐QTLs were significantly enriched in the biologically relevant tissues to their respective disease states, for example, in thyroid cancer, eRNA‐QTLs were significantly enriched in the relevant thyroid tissue (see Methods; logOR = 2.55, *P*‐value = 1.11 × 10^−14^; Figure S4E,F, Supporting Information), whereas eQTLs did not exhibit a similar pattern (logOR = 0.07, *P*‐value = 0.83). Likewise, mammary breast and Adipose‐Subcutaneous tissue are associated with breast cancer (Figure S4G,H, Supporting Information). To examine the proportion of eRNA‐QTLs associated with cancer heritability, we used a partitioned heritability analysis using stratified (S)‐ LD score regression (LDSC).^[^ ^39^, ^40^ ^]^ We observed that eRNA‐QTLs can contribute a median of 16.60% of heritability, compared to 24.40% for eQTLs (**Figure** **3**A). Notably, breast cancer exhibited the highest proportion of heritability contributed by eRNA‐QTLs (Figure 3A).

To systematically investigate whether cancer risk loci share the same causal variants as eRNA‐QTLs, we employed summary data‐based Mendelian randomization (SMR)^[^ ^41^ ^]^ and colocalization analyses.^[^ ^42^ ^]^ Our analysis revealed that 33 cancer GWAS traits across 13 cancer types colocalized with at least one type of molecular QTLs (Figure 3B; Tables S4 and S5, Supporting Information). Specifically, we identified 806 eQTLs and 228 eRNA‐QTLs that colocalized with cancer GWAS risk loci (Figure 3B). We further analyzed the proportion of co‐localized GWAS risk loci and found that eRNA‐QTLs co‐localized with a median of 28.48% of GWAS risk loci (Figure 3C). Interestingly, 70.02% of them were exclusively detected by eRNA‐QTL but not eQTL (Figure S5A, Supporting Information). For example, we identified eRNA‐QTL in *CLPTM1L* eRNA (*CLPTM1Le*) exhibited strong colocalization with cervical cancer risk loci (PP_H4_ = 0.92) in uterus tissue, but not with eQTL (PP_H4_ = 0.01; Figure 3D). *CLPTM1L* encodes cleft lip and palate transmembrane protein 1‐like and could increase susceptibility to various cancers. Notably, although *TERT* is also a nearby gene, it is not expressed in the uterus tissue (Figure S5B, Supporting Information); in addition, gene‐based transcriptome‐wide association study (eTWAS) analysis also indicates that *TERT* expression does not fully explain the GWAS risk loci of cervical cancer (*P*_Bonferroni_ = 0.99; Figure S5C, Supporting Information). In another instance, eRNA‐QTL rs62431527 in *CNR1* eRNA (*CNR1e*) exhibited strong colocalization with ovarian cancer (PP_H4_ = 0.99), whereas there was no colocalization with the eQTL of *CNR1* (PP_H4_ = 0.00; Figure 3E).

To further investigate whether eRNA‐QTLs are enriched at causal cancer risk loci, we conducted fine mapping on colocalized eRNA‐QTLs and compared them with cancer risk loci within a 95% credible set. Our analysis revealed that cancer‐credible set variants shared with eRNA‐QTLs had a significantly higher posterior inclusion probability (PIP) than those not shared with eRNA‐QTLs (Mann‐Whitney U test, *P* = 1.41 × 10^−6^; Figure 3F). For example, the eRNA‐QTL variant rs10941679, which resides within the breast cancer risk locus 5p12 for *FGF10e* (Figure 3G). In our fine‐mapping analysis, rs10941679 exhibited a robust PIP of 0.91 for being a causal variant implicated in breast cancer. In contrast, other variants falling within the identical cancer risk locus but lacking any linkage to eRNA‐QTLs exhibited significantly diminished PIP values for being causal variants for breast cancer (PIP < 0.1). Moreover, the inclusion of eRNA‐QTLs significantly improved the genetic resolution of cancer‐credible sets, leading to the identification of 19.58% risk loci with one potential causal variant compared with only 12.45% risk loci when eRNA‐QTLs were not considered (Figure 3H). These results suggest that eRNA‐QTLs contribute distinguishably to cancer risk variants.

### 2.5 Enhancer Transcriptome‐Wide Association Study Reveals Novel Cancer Susceptibility eRNAs

To systematically identify and prioritize candidate functional eRNAs associated with human cancers, we conducted a pan‐cancer eRNA‐based transcriptome‐wide association study (eRNA‐TWAS) using the data from 49 normal and 31 tumor tissues. This approach allowed us to directly explore the relationship between the genetically predicted eRNA expression and cancer risk. For each tissue, we employed a mixed‐linear model to estimate the heritability of eRNA expression based on SNPs located near the eRNA in reference cohorts with matched RNA‐seq and genotype data. Only eRNAs with significant heritability estimates (*P* < 0.05) were included in further analyses. We further trained prediction models using FUSION, including methods like best linear unbiased predictor (BLUP), elastic‐net regression (ENET), and lasso regression (LASSO). Cross‐validation was utilized to select the most accurate prediction model for each specific eRNA. In total, we generated 34 633 tissue‐specific eRNA‐TWAS prediction models covering 8498 unique eRNAs from both normal and tumor tissues. The number of prediction models was highly correlated with the sample size of the reference panels (Figure S6A, Supporting Information). The average in‐sample prediction accuracy for eRNA‐TWAS models was 80.45%, comparable to previous eTWAS models.^[^ ^43^ ^]^

**Figure 4:**
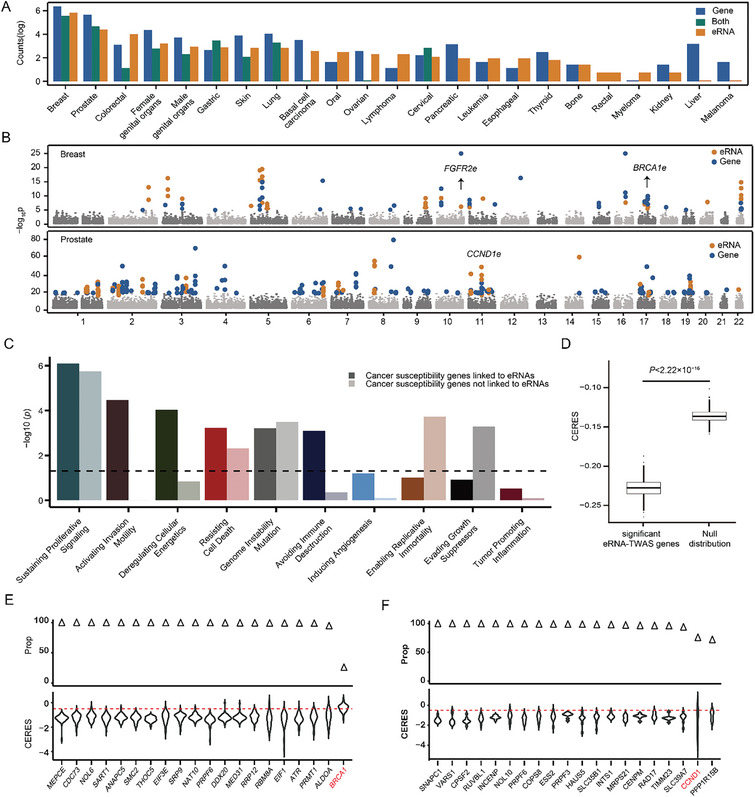
Landscape of cancer susceptibility eRNAs across 23 cancer types. (A) Cancer susceptibility eRNAs and genes were identified utilizing eRNA‐TWAS and eTWAS models. The color coding denotes cancer susceptibility genes as follows: blue for those identified solely by eTWAS, yellow for those identified solely by eRNA‐TWAS, and green for genes identified by both eTWAS and eRNA‐TWAS. (B) A Manhattan plot was meticulously constructed to visually represent TWAS findings for both breast and prostate cancer. eRNAs identified via eRNA‐TWAS are marked in yellow for easy visual identification, whereas genes uncovered through eTWAS are marked in blue. Each dot signifies the negative logarithm (to base 10) of the *P*‐value associated with each eRNA and gene identified through eRNA‐TWAS and eTWAS, respectively, plotted along the y‐axis. (C) Enrichment of cancer susceptibility eRNA‐linked genes and cancer susceptibility genes not linked to eRNAs in cancer hallmarks. We define cancer susceptibility genes identified exclusively through eTWAS as cancer susceptibility genes not linked to eRNAs. (D) Box plots displaying data from the Sanger DepMap Project Score highlighting cancer susceptibility eRNA‐link genes contributing to cell proliferation. In this context, ‘significant eRNA‐TWAS genes’ refers to eRNA‐linked genes identified as being associated with cancer through eRNA‐TWAS. Conversely, ‘Null distribution’ refers to eRNA‐linked genes unrelated to cancer. CERES scores were used to assess the essential levels of genes considering the computational effects of copy number and depletion of gene‐targeting guide RNAs. (E) Proportion of cell lines exhibiting CERES scores < −0.5 for genes associated with eRNA‐linked breast cancer susceptibility (top panel) and the top 20 cancer susceptibility eRNA‐linked genes with the lowest CERES scores evaluated in breast cell lines (bottom panel). *BRCA1*, a well‐established breast cancer susceptibility gene, was used as a positive control. Red dashes represent the median CERES score cutoff value of < −0.5, indicating a crucial role in cell proliferation. (F) Proportion of cell lines exhibiting CERES scores < −0.5 for genes associated with eRNA‐linked prostate cancer susceptibility (top panel) and the top 20 cancer susceptibility eRNA‐linked genes with the lowest CERES scores evaluated in prostate cell lines (bottom panel). *CCND1*, a well‐known oncogene in prostate cancer, was used as a positive control.

We applied our prediction models to 57 well‐powered GWAS summary statistics across 23 cancer types and identified 626 eRNAs and 1,011 eRNA‐linked genes significantly associated with cancer susceptibility (adjusted *P* < 0.05; **Figure** **4**A; Table S6, Supporting Information). Notably, 54.90% of eRNA‐linked genes were uniquely identified through eRNA‐TWAS and not by traditional gene expression studies (Figure 4A). For instance, within the eRNA‐TWAS‐identified breast cancer susceptibility eRNA‐linked genes, two widely recognized breast cancer susceptibility genes, *BRCA1* and *FGFR2* (Figure 4B), were found to have statistically significant associations (FDR < 0.05). Also, in the context of prostate cancer, *CCND1*, a well‐established gene associated with prostate cancer susceptibility, was also identified (Figure 4B). Additionally, there are 56 eRNAs and 145 eRNA‐linked genes that were identified both in the colocalization and eRNA‐TWAS analyses (Table S7, Supporting Information) and 6 eRNAs and 29 eRNA‐linked genes that were consistently identified in eRNA‐TWAS analyses for both GTEx and TCGA datasets (Table S6, Supporting Information). Interestingly, our eRNA‐TWAS identified multiple known cancer risk genes, such as GATA‐binding factor 6 (GATA6), which is significantly associated with breast cancer (*P*_eRNA‐TWAS_ = 3.69 × 10^−13^, *P*_eTWAS_ = 0.89). This finding suggests that eRNA expression of GATA6, rather than its gene expression, mediates breast cancer risk. We performed a cancer hallmark enrichment analysis on these putative eRNA‐linked cancer susceptibility genes. Our findings revealed that these genes were significantly enriched in several cancer hallmarks pathways (Figure S6B, Supporting Information),^[^ ^44^ ^]^ including sustaining proliferative signaling (*P* = 8.12 × 10^−7^; Figure 4C), activating invasion motility (*P* = 3.41 × 10^−5^), deregulating cellular energetics (*P* = 9.23 × 10^−5^), resisting cell death (*P* = 5.92 × 10^−4^).

To further explore the functional role of putative eRNA‐linked cancer susceptibility genes, we analyzed the effect of gene silencing on cell proliferation in cancer cell lines using CRISPR‐Cas9 gene essentiality screen data.^[^ ^45^ ^]^ We used the CERES score to represent gene essentiality levels, which adjusts for the computational effects of copy number variations and the depletion of gene‐targeting guide RNAs. In each cell line, a lower CERES score indicates a higher degree of gene essentiality. Our analysis revealed 193 eRNA‐linked genes demonstrated strong evidence of their essential roles in promoting cell proliferation (CERES score < ‐0.5). Moreover, the mean CERES scores of eRNA‐TWAS genes exhibited significantly higher essentiality in promoting cell proliferation compared to those non‐eRNA‐TWAS genes (Mann‐Whitney U test, *P* < 2.2**×**10^−16^; Figure 4D). For example, 19 genes exhibited comparable or even higher levels of essentiality than the well‐established breast cancer susceptibility gene *BRCA1* (*BRCA1*_CERESscore_ = −0.36; Figure 4E). Similarly, in prostate cancer, 18 eRNA‐linked genes associated with prostate cancer susceptibility displayed comparable or even higher levels of essentiality than the well‐known prostate cancer oncogene *CCND1* (*CCND1*_CERESscore_ = ‐1.20; Figure 4F). Notably, we observed a significant degree of essentiality in prostate cell lines for *SNAPC1*, a newly identified susceptibility eRNA target gene (*SNAPC1*_CERESscore_ = ‐1.46). We also noted that *SNAPC1* was found to be essential for the proliferation of all studied cell lines (N = 1,095; Figure 4F), whereas *CCND1* was essential for the proliferation of 75.53% of cell lines analyzed (N = 827; Figure 4F). Collectively, our eRNA transcriptome‐wide association analysis successfully identified extensive known and novel eRNAs with functional implications in multiple cancer types.

### 2.6 Experimental Validation of Prostate Cancer Novel Susceptibility eRNAs

**Figure 5:**
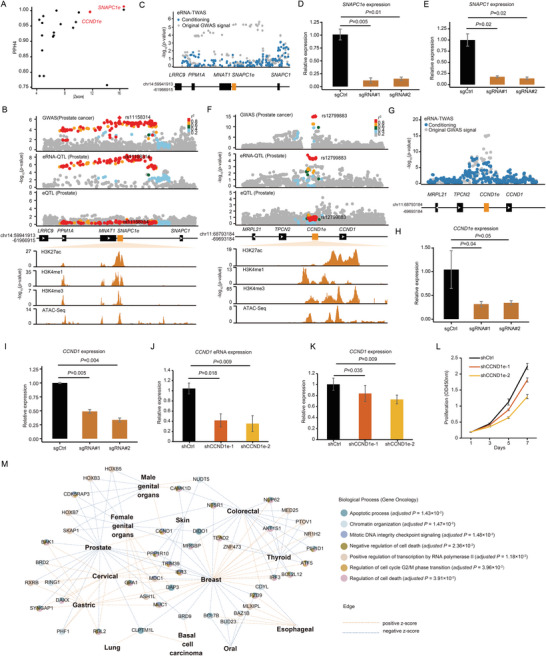
Validation of prostate cancer novel susceptibility eRNAs and integrative network analysis of cancer susceptibility eRNA‐link genes. (A) Correlation between the absolute effect sizes (z‐scores) as assessed by eRNA‐TWAS and the PPs (PP_H4_) derived from colocalization analysis for significant eRNAs linked to prostate cancer susceptibility. Points highlighted in red denote eRNAs with both high PIP in colocalization analysis and substantial effect sizes in eRNA‐TWAS, underscoring the eRNAs that exhibit strong evidence of association with prostate cancer susceptibility. (B) LocusZoom plot illustrating the association of prostate cancer GWAS SNPs, eRNA‐QTLs, and eQTLs at the *SNAPC1* eRNA (*SNAPC1e*) locus. SNPs are colored by LD (r2). Genome browser visualization highlights the landscape of histone modifications and ATAC‐seq peaks at the *SNAPC1e* locus in the PC3 cell line. Histone modification and ATAC‐seq data were obtained from the ENCODE Project, providing a comprehensive view of the regulatory elements influencing the *SNAPC1e* locus. (C) Manhattan plot depicting prostate cancer GWAS signals before (grey color) and after conditioning on *SNAPC1e* expression (blue color). (D‐E) Repression of *SNAPC1e* using Zim3‐KRAB‐dCas9 led to a significant reduction in the expression of their enhancer‐transcribed eRNAs (D) and target gene (E), as determined by quantitative PCR analysis in PC3 cells. This observation was statistically validated using a two‐sided t‐test, based on data from three independent experiments, with results presented as mean ± standard error. The term ‘sgRNA’ refers to single guide RNA. (F) LocusZoom plot illustrating the association of prostate cancer GWAS SNPs, eRNA‐QTLs, and eQTLs at the *CCND1* eRNA (*CCND1e*) locus. Genome browser visualization highlights the landscape of histone modifications and ATAC‐seq peaks at the *CCND1e* locus in the PC3 cell line (bottom panels). Histone modification and ATAC‐seq data were obtained from the ENCODE Project, providing a comprehensive view of the regulatory elements influencing the *CCND1e* locus. (G) Manhattan plot depicting cancer GWAS signals before (grey color) and after conditioning on *CCND1e* expression (blue color). (H‐I) Repression of *CCND1e* using Zim3‐KRAB‐dCas9 led to a significant reduction in the expression of their enhancer‐transcribed eRNAs (H) and target gene (I), as determined by quantitative PCR analysis in PC3 cells. (J‐K) Quantitative reverse transcription (qRT)‐PCR measuring indicated enhancer‐transcribed eRNA (J) and target gene (K) expression upon shRNA mediated CCND1e knockdown in PC3 cells. (n = 3). (L) Cell proliferation of shRNA mediated CCND1e knockdown cells was analyzed on days 1, 3, 5, and 7 (n  =  3). (M) Integrative network analysis of cancer susceptibility eRNA‐link genes. Each node in the network corresponds to a distinct eRNA‐linked gene, with the node's color indicating the specific pathway gene set to which it belongs. The edges connecting the nodes are color‐coded: blue edges signify a negative Z‐score in eRNA‐TWAS, suggesting that reduced eRNA expression is associated with an elevated cancer risk, while yellow edges denote a positive Z‐score, indicating that increased eRNA expression is linked to a higher cancer risk.

Our colocalization and eRNA‐TWAS results identified multiple novel cancer‐susceptibility eRNAs. Among these candidates, we focused on two novel eRNAs for further experimental validation, as they were identified in both colocalization and eRNA‐TWAS analysis (**Figure** **5**A). We identified an eRNA‐QTL in *SNAPC1e* that exhibited strong colocalization with prostate cancer risk loci, but not with eQTL (Figure 5B). Furthermore, *SNAPC1e* was conditionally independent at the associated prostate cancer loci (Figure 5C), implying that eRNA‐mediated risk variants substantially explain the GWAS signal within this genomic region. To functionally assess the role of eRNA in tumor cells, we employed CRISPR interference (CRISPRi) to specifically suppress the enhancer activity of *SNAPC1e* in PC3 prostate cancer cells. This targeted suppression resulted in a notable decrease in the expression levels of the enhancer‐derived eRNA, *SNAPC1e*, and its target gene, *SNAPC1* (Figure 5D,E). We also used *SNAPC1e* shRNA to further verify the role of *SNAPC1e* in cell proliferation. Although a shRNA with mild knockdown efficiency was applied, it also resulted in a similarly modest decrease in prostate cancer cell proliferation rate (Figure S6C–E, Supporting Information). Furthermore, we observed more compelling results with the eRNA‐QTL in *CCND1e*, which also exhibited strong colocalization with prostate cancer risk loci, but did not colocalize with the eQTL of *CCND1* (Figure 5F). Moreover, *CCND1e* was conditionally independent at the associated prostate cancer loci (Figure 5G), further supporting the notion that eRNA‐mediated regulation at this locus is a key driver of the GWAS signal. To functionally assess the role of eRNA in tumor cells, we employed CRISPRi to suppress the enhancer activity of *CCND1e* in PC3 prostate cancer cells, which resulted in a marked decrease in the expression of both *CCND1e* and its target gene, *CCND1* (Figure 5H,I). We also used shRNA to knockdown *CCND1e* to further validate its role in cell proliferation. As expected, the knockdown of *CCND1e* consistently resulted in the significant downregulation of *CCND1* expression as well as cell proliferation rate (Figure 5J,K). These findings underscore the critical role of *CCND1e* in the regulation of *CCND1* expression and highlight its potential as a therapeutic target in prostate cancer treatment. In conclusion, our comprehensive analysis integrating colocalization and eRNA‐TWAS techniques successfully identified numerous novel cancer susceptibility eRNA‐linked genes. These findings highlight the critical role of enhancer RNAs in cancer biology and provide a foundation for further exploration of their therapeutic potential.

## 3 Discussion

GWAS has been highly effective in identifying thousands of SNPs associated with a wide range of complex human traits and diseases. Notably, most SNPs identified through GWAS are located within noncoding regions of the genome, particularly enhancer regions that play a crucial role in regulating gene expression. Despite these findings, the biological relationship between genetic variants and disease susceptibility has remained largely unknown. A promising strategy to bridge this gap involves identifying eQTLs as essential intermediaries linking GWAS variant signals with disease phenotypes. eQTLs may provide valuable insights into how genetic variants contribute to the development and progression of diseases, whereas they can only explain a small proportion of disease‐related variants.

Here, we conducted QTL mapping for expressed eRNAs utilizing 49 normal tissues from the GTEx and 31 tumor tissues from the TCGA. Our analysis identified 11,757 eRNA‐QTLs within the normal tissues and 9,316 eRNA‐QTLs within the tumor tissues. Our CRISPR‐based base editing experiment confirmed that disruption of eRNA‐QTLs affects the binding motifs of TFs and modifies the expression of eRNAs. eRNA‐QTLs exhibited greater tissue specificity than eQTLs. The large‐scale eRNA‐QTL atlas allowed the generation of a genetic map between regulatory variants and complex human traits. By performing SMR and colocalization analyses, we revealed that 70.02% of colocalization events were unique to eRNA‐QTLs and not shared with eQTLs. Our eRNA‐TWAS analyses identified 626 eRNAs associated with cancer susceptibility across 23 cancer types. Notably, 54.90% of the target genes of these eRNAs were overlooked by traditional gene expression studies. We experimentally validated that the inhibition of two newly identified susceptibility eRNAs (*CCND1e* and *SNAPC1e*) leads to a reduction in the expression of their target genes which ultimately contributes to cell proliferation. Our findings provide additional evidence for the oncogenic potential and therapeutic liability of eRNAs. This also suggests that eRNA‐QTLs can explain additional disease signals that eQTLs ignore. To gain a deeper understanding of the functional interactions and relationships among these eRNA‐linked genes, we conducted an integrative network analysis and revealed that cancer susceptibility eRNA‐linked genes strongly converge in shared pathways, including mitotic DNA integrity checkpoint signaling (Figure 5M; hypergeometric test, adjusted *P* = 1.48 × 10^−3^) and the apoptotic process (hypergeometric test, adjusted *P* = 1.43 × 10^−2^). Overall, these results demonstrate that eRNA‐TWAS can effectively identify cancer susceptibility eRNAs and their linked genes that are essential for cancer cell proliferation across various cancer types.

Notably, a single eRNA only partially contributes to target gene expression, and multiple eRNA interactions can influence target gene expression levels.^[^ ^46^ ^]^ For example, we discovered that the disease locus associated with inflammatory bowel disease showed colocalization with the *PSMG1* eRNA (*PSMG1e*) signal (Figure S7A, Supporting Information). However, this colocalization could not be explained by the expression of the target gene *PSMG1*. Our SMR analysis further supports these findings, indicating that the inflammatory bowel disease locus could be explained, at least partially, by the role of *PSMG1e* (Figure S7B, Supporting Information). Examining single‐cell level expression, we observed that *PSMG1e* was only expressed in a specific cluster, whereas its target gene *PSMG1* was expressed across all clusters (Figure S7C, Supporting Information). These single‐cell expression data provide additional evidence of the differential expression patterns between *PSMG1e* and its target genes, highlighting the complexity of their regulatory relationships in specific cellular contexts. Considering the complexity of eRNA regulation, the eRNA‐QTL strategy is a powerful approach to dissecting eRNA targets and their potential roles in disease‐related processes.

In our comprehensive analysis of eRNA‐mediated genetic effects, we have identified a significant class of pan‐cancer susceptibility eRNAs and constructed an extensive atlas of eRNA‐QTLs. Our findings underscore the essential role of eRNAs in modulating cancer risk and provide a robust framework for understanding the genetic architecture of cancer susceptibility. It is worth noting that our study builds upon and extends the work of Han et al.,^[^ ^47^ ^]^ who systematically investigated the impact of genetic variants on eRNA expression using large‐scale omics data from TCGA. One of the key distinctions in our study is the focus on the functional characterization of cancer risk loci through eRNA‐TWAS. While Han et al. provided a broad overview of eRNA‐QTLs and their implications in cancer, we have delved deeper into the specific mechanisms by which eRNAs modulate cancer risk. Our identification of 626 significant cancer susceptibility eRNAs, including 54.90% of eRNA target genes not identified by traditional gene expression studies, highlights the unique contribution of our study. Furthermore, our experimental validation of the enhancer functionality of two novel susceptibility eRNAs, *CCND1e* and *SNAPC1e*, through CRISPR inhibition, demonstrating their role in regulating target gene expression and cancer cell proliferation.

In our investigation into the eRNA‐mediated genetic effects across various human tissues and cancer types, we have unveiled a significant class of pan‐cancer susceptibility eRNAs. Despite the substantial findings of this study, it is imperative to consider the inherent limitations, particularly those related to the detection of eRNAs. One critical limitation is that polyA RNA‐seq captures only a subset of polyadenylated eRNAs due to their transient nature and the limitation of polyA selection. Moreover, non‐polyadenylated eRNAs that may be critical to cancer etiology are likely underrepresented in our dataset. To mitigate these challenges, future research should leverage alternative sequencing approaches such as GRO/PRO‐cap^[^ ^19^, ^48^ ^]^ specifically designed to comprehensively detect labile eRNA species in the population cohorts. Despite these limitations, our study provides a robust foundation for further exploration of the role of eRNAs in cancer susceptibility. As sequencing technologies and analytical tools evolve, we anticipate a more precise understanding of how eRNAs contribute to gene regulation and disease pathogenesis.

In summary, eRNA‐QTL analysis provides a more comprehensive understanding of genetic regulation and disease mechanisms in cancer risk beyond what traditional eQTL analysis can capture. It expands the current strategy of molecular QTLs and supports the hypothesis that eRNA‐QTLs contribute to a range of human phenotypes by regulating gene expression in a cell type‐specific manner.

## 4 Experimental Section

### Study Subjects

A comprehensive dataset comprising 28 033 RNA sequencing samples from 11 606 individuals is employed. This dataset included 838 genotype datasets and 17 265 genotype‐matched RNA‐seq datasets across 49 human normal tissues sourced from the GTEx project.^[^ ^20^ ^]^ Additionally, 10 768 genotype‐matched RNA‐seq datasets were incorporated across 31 human tumor tissues obtained from the TCGA dataset.

### eRNA Annotation

ENCODE, FANTOM, and Roadmap Epigenomics datasets were integrated to annotate enhancers. Enhancers that appeared in at least two datasets were considered. Similar to previous study,^[^ ^9^ ^]^ a 3‐kb region around the center of the enhancer was defined as an eRNA region. The initial collection comprises 45 411 eRNAs, which include 7879 previously annotated lncRNAs derived from enhancer regions. Instead, only tRNAs, snoRNAs, and miRNAs, following a similar strategy as recently described were excluded.^[^ ^9^ ^]^ To avoid the potential interference of known transcripts, only intergenic eRNAs that did not overlap with existing annotations, including protein‐coding RNAs and non‐coding RNAs (ncRNAs), were retained.

### GTEx Data Collection and Quality Control

A comprehensive dataset was acquired, comprising RNA‐seq BAM files from 17 382 human normal samples across 54 tissues in 948 individuals obtained from the GTEx project (dbGaP, phs000424.v8.p2).^[^ ^20^ ^]^ To align the original RNA‐seq reads to the Human Reference Genome Build GRCh38 (hg38), STAR^[^ ^49^ ^]^ was employed following the alignment parameters specified in the GTEx study.^[^ ^20^ ^]^ Rigorous measures were taken to ensure the integrity of the data, including the exclusion of BAM files generated from diseased tissues and tissue types with limited sample sizes. To maintain consistency and reliability, RNA‐seq BAM files lacking genotype data were also removed from the analysis freeze, as they were not included in the GTEx study.

Genotype information was derived from whole‐genome sequencing data obtained from the GTEx v8 release.^[^ ^20^ ^]^ Burrows‐Wheeler alignment^[^ ^50^ ^]^ was utilized to align whole‐genome sequencing reads to the Human Reference Genome Build GRCh38 (hg38), and GATK HaplotypeCaller v3.5 was employed to call variants in variant call format. Subsequently, a stringent quality control process implemented by the GTEx Consortium led to the exclusion of low‐quality samples, resulting in a final analysis freeze set that encompassed variants called from 838 donors. To enrich the dataset further, imputation and phasing techniques using SHAPEIT v2^[^ ^51^ ^]^ were applied to the final variants. Additionally, sample description files providing valuable contextual information were downloaded from the GTEx Portal (https://www.gtexportal.org) and associated with the analyzed samples. To further identify hidden genetic relationships and sample duplications, identity‐by‐descent (IBD) between all sample pairs was calculated. Only samples with IBD < 0.25 were retained. SNPs were excluded if they: 1) had a low call rate (< 95%), 2) had a low minor allele frequency (MAF < 1%), and 3) were out of Hardy–Weinberg equilibrium (*P* < 1 × 10^−8^). Samples with a low call rate (< 95%) were excluded. The eQTL data from normal and tumor tissues were downloaded from the GTEx project^[^ ^20^ ^]^ (https://www.gtexportal.org) and PancanQTL^[^ ^52^ ^]^ (https://gong‐lab.hzau.edu.cn/PancanQTL/), respectively.

### TCGA Data Collection, Imputation, and Quality Control

A comprehensive dataset comprising ≈11 000 human tumors was obtained across 31 diverse cancer types from the legacy archive of TCGA^[^ ^53^ ^]^ (https://portal.gdc.cancer.gov/legacy‐archive). The RNA‐seq raw data underwent processing by the TCGA consortium. STAR^[^ ^49^ ^]^ was utilized to align the RNA‐seq data to the Human Reference Genome Build GRCh38 (hg38).

The genotype data obtained from the TCGA legacy archive were genotyped using Affymetrix Genome‐Wide SNP 6.0 arrays. Birdseed genotyping files containing information on ≈905 600 variants across a cohort of approximately 11 000 samples were downloaded for further analysis. Genotype data were aligned to the Human Reference Genome Build GRCh38 (hg38). IMPUTE2 software was utilized to impute genetic variants for the TCGA samples. The imputation process used the 1000 Genomes Project as a reference panel. To ensure the reliability of the imputed data, stringent quality control measures were applied. Specifically, variants with an imputation confidence score (INFO) ≥ 0.8, MAF ≥ 1%, SNP missing rate < 5%, and Hardy–Weinberg equilibrium *P* > 1 × 10^−8^ were considered. These strict criteria were employed as a cutoff to retain only high‐confidence SNPs for subsequent analyses.

### eRNA Quantification

Quantification of eRNA expression was conducted through eRNA annotation. Reads per million was applied to quantify eRNA expression and then the inverse‐normal transformation method was used for normalization. Only eRNAs with ≥ 1 reads per million were kept for subsequent analyses.

### eRNA‐QTL Mapping

eRNA‐QTL analysis was conducted with QTLtools version 1.2.^[^ ^23^ ^]^ A linear regression model was employed to adjust for covariates and the correct mode was used to regress out covariates from GTEx and TCGA sample expression data (Figure S1B,C, Supporting Information). The molecular phenotype data obtained from RNA sequencing had variability from biological and technical factors. To address technical variability while preserving biological variability, three types of covariates were accounted: 1) sex, age, body‐mass index, PCR, and platform for GTEx and age, gender, tumor grade, and stage for TCGA using the metadata provided in GTEx^[^ ^20^ ^]^ and TCGA,^[^ ^53^ ^]^ respectively; 2) the first five genotype principal components (PCs) derived from individuals’ genotypes to correct for population stratification observed between samples; and 3) phenotypes PCs. We performed phenotype PC analysis using QTLtools software's ‘pca’ mode to capture experimental/technical variability by centering and scaling the expression data. To determine the optimal number of phenotype PCs capturing technical variability for eRNA‐QTL discovery in GTEx and TCGA samples, we conducted multiple rounds of eRNA‐QTL mapping. In each iteration, we integrated PCs derived from phenotypes, sequentially introducing 0, 5, 10, 20, 30, 40, 50, 60, 70, and 80 PCs as covariates for phenotype data. Utilizing this stepwise and iterative approach, the set of PCs that optimized the detection of eRNA‐QTL associations within the GTEx and TCGA cohorts was meticulously identified. This strategy ensured the enhancement of eRNA‐QTL discovery by systematically accounting for phenotypic variability (Figure S1C, Supporting Information). The optimal number of PCs employed in the final eRNA‐QTL analysis is presented in Table S1 (Supporting Information).

For eRNA‐QTL testing, permutations with 1000 repetitions were performed to establish the null distribution of associations for each eRNA individually. Using the q‐value package in R,^[^ ^54^ ^]^ the FDR < 0.05 for multiple tests was achieved. Moreover, the quantifications were rank‐normalized on a per‐phenotype basis across all samples using the –normal option in QTLtools, ensuring a normal distribution with a mean of 0 and standard deviation of 1 (N (0, 1)). Nominal *P*‐values for all SNP‐eRNA pairs within the cis‐window (1M bp) were obtained with the nominal pass implemented in the QTLtools package. Associations between SNPs and eRNAs reaching the significance threshold corresponding to the FDR < 0.05 were retained for further analysis.

### Conditional eRNA‐QTL Discovery

To evaluate the influence of additional genetic variants on the expression of a specific eRNA, a conditional analysis for each eRNA was conducted using the “‐mapping” option and a forward‐backward stepwise regression implemented in QTLtools.^[^ ^23^ ^]^ The inclusion of these factors as covariates in the QTL mapping model enabled the assessment of whether an observed QTL was independent of other genetic factors.

### GWAS Summary Statistics Curation and Integration

GWAS summary statistics from various sources (Table S3, Supporting Information)were collected, including published literature, the UK Biobank Imputed Dataset v.3, FinnGen Biobank, and JENGER, as these datasets were publicly accessible. The selection of studies was based on the availability of the original publications, clear recording of population‐related information, and adequate sample sizes. To avoid duplication, we identified and retained the dataset with the most comprehensive information whenever redundancy occurred across different sources. We extracted essential details such as sample size, population, and data sources from the original studies.

The analysis focused only on GWAS data with population information precisely mapped to European ancestry individuals. To ensure data quality, we excluded individuals from consideration if their sample sizes were < 50 000, and any studies that potentially had duplicated patients or controls were removed. To assess the quality of the remaining GWAS summary statistics, a thorough examination was conducted using the R package xQTLbiolinks.^[^ ^55^ ^]^ This involved analyzing quantile–quantile (QQ)‐plots to identify any inflation issues and P–Z plots to evaluate the consistency of analytical parameters such as beta values, standard errors, and *P*‐values. Following this rigorous quality check, 57 cancer GWAS summary statistic datasets deemed suitable were identified for further downstream analyses (Table S3, Supporting Information). To ensure consistency of the cancer GWAS summary statistics, which were based on different genome versions, CrossMap was utilized.^[^ ^56^ ^]^ This tool was employed to convert the GWAS coordinates to the Human Reference Genome Build GRCh38 (hg38), harmonizing the GWAS data.

### Heritability Estimation

A restricted maximum‐likelihood model implemented in GCTA^[^ ^27^ ^]^ was used to estimate the total heritability of eRNAs arising from common genetic variants (MAF > 0.01). Heritability, in this context, refers to the proportion of phenotypic variation that can be attributed to the total genetic variation across all assessed loci. To estimate total genetic variation, GCTA generates a ‘genetic relatedness matrix’ that captures overall genetic dissimilarities among individuals in the study cohort. This comprehensive approach provided insights into the contribution of genetic factors to the observed variation in eRNA expression levels, shedding light on the heritability of these regulatory elements.

### Evaluation of eRNA‐QTL Sharing Between Tissues

To identify patterns of tissue sharing and tissue specificity, masher analyses were performed using a multivariate adaptive shrinkage approach implemented in the R package mashR.^[^ ^28^ ^]^ mashR computes posterior estimates of eRNA‐QTL effect sizes and standard errors across tissues with multi‐tissue eRNA‐QTL summary statistics. Tissue sharing is defined as an effect size within a factor of 0.5 in the same direction. Tissue specificity was described as a local false sign rate < 0.05 and z‐score of at least a twofold difference.

### eRNA‐QTL Enrichment in Genomic Annotations

To gain insights into the functional enrichment of identified eRNA‐QTLs, functional enrichment analyses were performed using torus software^[^ ^29^ ^]^ following a similar approach as the GTEx Consortium. To perform the annotations, datasets from various sources were used. ENCODE provided gene regulatory elements and open chromatin annotations, and Ensembl contributed gene body annotations. Chromatin state predictions were obtained from ROADMAP, and CpG island annotations were collected from the UCSC Genome Browser. For each specific tissue, the torus analysis generated enrichment estimates in point estimates derived using maximum‐likelihood estimation. These estimates represent the logarithm of the odds ratio. To evaluate enrichment across multiple tissues, a random‐effects model was employed to model the single‐tissue enrichment estimates (i.e., log of odds ratio). A false discovery rate control was employed with a significance threshold set at 5% for multiple test correction.

### Mediation Analysis for SNP‐eRNA‐Gene Triplets

In the context of mediating gene expression via eRNAs, mediation analyses were conducted for each X‐M‐Y triplet. Here, X denotes a genetic variant significantly associated with both eRNA and gene expression levels; M represents the eRNA expression; and Y signifies the gene expression. The mediation analysis was performed using the bmediatR^[^ ^32^ ^]^ package, incorporating covariates pertinent to eRNA‐QTL and eQTL data to account for population structure and technical factors. Three distinct network topologies relevant to the hypotheses being tested were focused on (Figure 2B), which included: 1) the complete mediation, in which the effect of a genetic variant on a phenotype Y is completely explained by the effect of the same variant on a phenotype M; 2) the partial mediation, in which the effect of a genetic variant on a phenotype Y is partially explained by the effect of the same variant on a phenotype M; and 3) the co‐local, in which the genetic variant independently influences both the gene and eRNA. To build the X‐M‐Y triples with biological links, the target genes of eRNAs were identified through co‐expression analysis, thereby linking eRNAs with genes. In order to obtain the most reliable results and reduce computing time, bmediatR was applied for the X‐M‐Y triplets where the genetic variant had the smallest *P*‐value in at least one phenotype using the covariates for eQTL and eRNA‐QTL mapping. Importantly, the relationship was considered as complete mediation if the posterior probability of this complete model was higher than 0.5. The same criterion was applied to the partial mediation and co‐local.

### Enrichment of eRNA‐QTLs in TFBSs

To examine the enrichment of TFBSs in eRNA‐QTLs and eQTLs, ChIP‐seq data for 194 TFs and 17 histone marks were analyzed from the ENCODE project, constructing 2 × 2 contingency tables for each TF. QTL variants were compared to a null distribution of similar variants lacking regulatory associations. This null distribution was generated by sampling 1000 random regulatory genetic variants for each QTL variant, matching them based on the relative distance to the transcription start site and minor allele frequency (within 1%). Only those variants were included that were not QTLs for any other eRNA or gene (nominal *P*‐value > 0.05). Enrichment for TFBSs was calculated as the proportion of regulatory associations within that TFBS compared with all regulatory variants relative to the same proportion in the null distribution of variants. The *P*‐value for this enrichment was determined using a Fisher exact test. To account for multiple tests, we applied an FDR threshold of < 0.05 using the ‘p.adjust’ function in R programming language.

### Fine Mapping of GWAS Loci

Fine‐mapping analysis was conducted on the curated cancer GWAS summary statistics using ancestry‐matched linkage disequilibrium (LD) information. A recent toolkit was utilized that integrates three fine‐mapping methods: PAINTOR (v.3.0), CAVIARBF (v.0.2.1), and FINEMAP (v.1.3.1). Each causal block was constrained to contain only one causal variant, and we applied the recommended parameters for these tools. These fine‐mapping methods yield the PIP of each variant as the causal one within a specified model. Subsequently, credible sets consisting of variants were identified with cumulative posterior inclusion probability values surpassing the 95% threshold.

### Enrichment of eRNA‐QTLs and eQTLs Within Cancer GWAS Risk Loci

GARFIELD (v2)^[^ ^38^ ^]^ was employed to investigate the enrichment of molecular QTLs (nominal *P*‐value < 1 × 10^−5^) within cancer GWAS risk loci. GARFIELD is a functional enrichment analysis tool that integrates GWAS findings with regulatory and functional annotations, while accounting for linkage disequilibrium, minor allele frequency, and proximity to transcription start sites.^[^ ^38^ ^]^ Additionally, different molecular QTLs (nominal *P*‐value < 1 × 10^−5^) were extracted that also belonged to cancer GWAS SNPs and quantile–quantile plots (Q–Q plots) were used to visualize the GWAS *P*‐values for those SNPs. These plots provided a useful visual representation to assess deviations from the expected null distribution, aiding in the identification of potential associations. To further explore the enrichment of heritability attributed to eRNA‐QTLs and eQTLs within GWAS risk loci, stratified LD score regression (v1.0.1) was applied to the cancer GWAS summary statistics. The analysis integrated functional categories into the ‘baseline‐LD model’ encompassing 53 additional functional categories. Distinct binary annotations were created for eRNA‐QTLs and eQTLs, with a value of 1 assigned to the most significant eRNA‐QTLs and eQTLs, whereas the remaining SNPs were assigned a value of 0. LD scores for the SNPs were computed using genotype data from individuals of European ancestry obtained from the 1000 Genome Project (phase 3), utilizing a window size of 1 cM. Ultimately, the heritability enrichment of each category was calculated by comparing the proportion of heritability explained by the category to the proportion of SNPs within that category.

### SMR Analysis

SMR analysis is a statistical method used in genetic epidemiology to investigate the causal relationship between a phenotype or trait and molecular phenotype. It involves the integration of summary‐level data from GWAS and QTL studies to examine whether the effect of a genetic variant on the phenotype is mediated through molecular phenotypes. SMR analysis helps identify potential causal relationships among genetic variants, molecular phenotypes, and complex traits. Here, SMR analysis was conducted to test whether the effect of a common genetic variant on a phenotype was mediated by eRNA expression. Further the heterogeneity in dependent instruments (HEIDI) test was performed to detect the existence of LD in the genetic association. *P*_HEIDI_ < 0.05 indicates that the observed genetic association could be due to LD between SNPs. The significance threshold of SMR was set at *P*_SMR_ < 0.05/N and *P*_HEIDI_ > 0.05, with N indicating the number of tests.

### Colocalization Analyses

A Bayesian method for colocalization analysis to determine whether there is shared causal genetic variation between molecular traits (e.g., eRNA expression) and a disease trait. Specifically, the R package ‘coloc’^[^ ^42^ ^]^ was used to assess the colocalization of cancer GWAS summary statistics and eRNA‐QTL and eQTL signals. The coloc package calculates five hypotheses: H0 (no association), H1 (GWAS association only), H2 (eRNA‐QTL or eQTL association only), H3 (both associations but not colocalized), and H4 (both associations and colocalized). Separate analyses were performed for each cancer risk phenotype and each proximal eRNA or gene using default parameters. To determine whether an eRNA or gene and GWAS signal were colocalized, the threshold of the PP_H4_ was set to be > 75%. Additionally, the ratio of PP_H4_ was required to the sum of PP_H3_ and PP_H4_ (PP_H4_/(PP_H3_ + PP_H4_)) to be ≥ 0.9. These criteria helped identify cases where there was strong evidence for both the eRNA or gene and GWAS associations being driven by the same underlying causal variants. LocusZoom (v.1.4) was employed to visualize regional plots and PLINK (v.1.90) to assess the LD between the identified causal SNP and other SNPs.

### eRNA‐TWAS for Cancer GWAS

The FUSION framework^[^ ^43^ ^]^ was utilized to perform eRNA‐TWAS. The approach began by employing a mixed‐linear model to estimate the heritability of the eRNA region. This estimation was based on SNPs with a MAF > 0.01 located within 1 Mb of the eRNA region using a reference panel that consisted of cohorts with matched RNA‐seq and genotype data. To ensure robust covariate adjustment, well‐established factors utilized in the QTL mapping section were incorporated to determine eRNA expression. Subsequently, only eRNAs with significant heritability estimates (*cis*‐h^2^) below a Bonferroni‐corrected *P*‐value of 0.05 were retained for further analysis. Within the FUSION framework, three different models were selected for weight calculation: BLUP, ENET, and LASSO. A cross‐validation approach was employed to determine the model with the optimal eRNA‐TWAS prediction accuracy for each gene. Subsequently, these eRNA‐TWAS prediction models were applied to GWAS summary statistics using the FDR threshold of 0.05. For the purpose of comparative analysis, gene‐based transcriptome‐wide association study (eTWAS) utilizing identical GWAS summary statistics was additionally performed. The gene expression TWAS models from the GTEx and TCGA project were sourced via PredictDB.^[^ ^57^ ^]^

### The Identification of eRNA‐Link Gene

Gene annotations were obtained from ENSEMBL (https://jul2023.archive.ensembl.org/index.html), GENCODE (https://www.gencodegenes.org/human/release_38.html), and UCSC (https://genome.ucsc.edu/index.html) and integrated them. The expression matrix of these genes across human tissues was collected from the GTEx portal, and across cancer types from TCGA. Putative target genes of eRNAs were identified by assessing proximity (≤ 1MB) and co‐expression (Spearman's correlation coefficient Rs ≥ 0.8 and adjusted *P* < 0.05) between individual eRNAs and their target genes across various tissues and cancer types. To ensure accuracy, eRNAs located within intronic regions of the target genes were excluded from the correlation analysis.

### Annotation of Cancer Susceptibility eRNA‐Link Genes in Cancer‐Relevant Gene Databases

To ascertain the intersection between the cancer susceptibility eRNA‐link genes and established cancer‐related genes, cancer‐related gene sets from reputable sources, including the Molecular Signatures Database (MSigDB), DORGE, and Catalogue of Somatic Mutations in Cancer (COSMIC) Gene Census (https://cancer.sanger.ac.uk/census) were compiled. Putative cancer‐related genes were identified by annotating them with specific key phrases, such as ‘breast cancer’ and ‘prostate cancer’.

### Gene Set Enrichment Analysis

Pan‐cancer identified cancer susceptibility eRNA‐link genes, filtering for those that encode HLA genes within MHC regions were selected. Gene set enrichment analysis (GSEA) was performed between the two groups to identify significantly altered Gene Ontology (GO) pathways utilizing GSEA software (v4.3.2).^[^ ^58^ ^]^ GSEA is a widely utilized software package that utilizes gene sets to discern different biological functions between two groups. GO analysis encompasses biological processes, cellular components, and molecular functions. The statistical significance of enrichment was determined using the hypergeometric distribution. *P‐*values generated by hypergeometric tests were FDR‐corrected for multiple testing, and a *P* < 0.05 was considered statistically significant. The integrative network graphs were visualized using Cytoscape, a software tool for visualizing and analyzing biological networks.

### Cell Line Maintenance and Generation

HEK293T cells were cultivated in Dulbecco's Modified Eagle Medium (Gibco, cat #: C11995500BT) supplemented with 1% penicillin/streptomycin and 10% fetal bovine serum (FBS; Gibco, cat #: C10010500BT), 100 IU mL^−1^ penicillin, and 100 µg mL^−1^ streptomycin (Gibco, cat #: 15140‐122) and were incubated in 5% CO_2_ at 37 °C. HEK293T cells were seeded onto 6‐well plates and transfected with BE4max‐NG or SpRY‐BE4max and sgRNA plasmids using EZtrans (Shanghai Life iLab Biotech, cat #: AC04L099) according to the manufacturer's instructions. Synthesized oligos (Table S8, Supporting Information) were annealed and ligated into the Bsa1‐digested pGL3‐U6‐pGK‐puro vector. To screen cells expressing pGL3‐U6‐sgRNA‐pGK‐puro, puromycin (2.5 µg mL^−1^; Merck, cat #: 540411) was added to cells after transfection for 24 h and then collected at 48 h post‐treatment. Transfected cells with high mutation efficiency were seeded onto a 96‐well plate with about 60 cells. After 12 days of cultivation, single clones were subjected to DNA extraction and genotyping. Clones with expected mutations were passaged for subsequent experiments.

PC3 cells obtained from the Cell Resource Center of Shanghai Institutes for Biological Sciences (Chinese Academy Science, Shanghai, China) were cultured in RPMI‐1640 (Gibco, cat #: C22400500BT) supplemented with 10% FBS, 100 IU mL^−1^ penicillin, and 100 µg mL^−1^ streptomycin. Lentivirus was produced by cotransfecting HEK293T cells with transfer plasmids and standard packaging vectors pMD2.G and psPAX2 using PEI according to the manufacturer's instructions. To generate PC3 cells stably expressing CRISPRi effectors, PC3 cells were infected with lentivirus containing Zim3‐KRAB‐dCas9 (pLX303) at low multiplicity of infection followed by 3 days puromycin selection.

### Genomic DNA Extraction and Genotyping

Transfected HEK293T cells were treated with lysis buffer consisting of 1.5 mM MgCl2, 10 mM Tris‐HCl (pH 8.0), 50 mM KCl, 0.5% Tween‐20, 0.5% Nonidet P‐40, and 100 µg mL^−1^ proteinase K (ThermoFisher Scientific). DNA fragments containing the targeting sites amplified by PCR with Phanta Max Super‐Fidelity DNA polymerase (Vazyme; P505) were subjected to Sanger sequencing for genotyping. The primers used for PCR are listed in Table S8 (Supporting Information).

### Assessing Transcriptional Effects via Targeted Enhancer Repression

Two guide RNA oligos per enhancer were inserted into the pBA900 (pU6‐sgRNA EF1Alpha‐puro‐T2A‐BFP) at the Blp1 and BstX1 restriction sites. gRNA constructs and control vector were separately packaged into lentivirus, which was subsequently transduced into PC3 cells stably expressing Zim3‐KRAB‐dCas9. Ten days post‐infection, transduced cells were sorted via flow cytometry using a BD FACS Aria3. Total RNA was extracted using the Quick‐RNA Miniprep Kit (cat#: R1055; Zymo Research), and cDNA was generated using the Hifair III 1st Strand cDNA Synthesis Super Mix for qPCR (gDNA digester plus) kit (Yeasen, cat #: 11141ES60). Quantitative PCR was performed using the Hieff qPCR SYBR Green Master Mix (Yeasen, cat #: 11203ES08) on a CFX96 machine (BIO‐RAD, Hercules, CA, USA). Experiments measuring the expression of each gene were repeated at least three times, with GAPDH used as the internal reference for expression. The gRNA oligos and primers used for qPCR are listed in Table S8 (Supporting Information).

### Generation of Stable Knockdown Cell Line Using Lentivirus Delivered shRNA

shRNAs against each eRNA were used to clone into pLKO.1‐puro vector (Table S8, Supporting Information). They were separately packaged into lentivirus, which was subsequently transduced into PC3 cells. Puromycin was applied to kill non‐infected cells 36 to 48 h after infection. After 2 days of selection, when non‐infected control cells were all dead, surviving cells were split and maintained with the same concentration of puromycin. After 3 days, cells were collected for RNA and tested by RT‐qPCR to confirm the successful shRNA knockdown efficiency of target genes.

### Cell Viability and Proliferation Assays for shRNA‐Mediated Knockdown

Cells were trypsinized, resuspended at 0.8 × 10^4^ cells mL^−1^, and seeded in 96‐well plates, with each well containing 100 µL medium of 0.8 × 10^3^ cells. Cell viability and proliferation were determined using CCK8 assays (Yeasen, cat#: 40203ES76) at designated time points by measuring the absorbance at 450 nm, following the manufacturer′s instructions. Values were obtained from four replicate wells for each treatment and time point. Results are representative of three independent experiments.

### RNA‐seq and Analysis

Total RNA extracted from HEK293T cells was subjected to strand‐specific RNA sequencing using the Illumina nova seq 6000 platform at Berry Genomics Co, Ltd (Beijing, China). Briefly, strand‐specific RNA‐seq libraries were prepared by combining the Ribo‐Zero rRNA Removal Kit (Epicentre, Madison, WI, USA) and the dUTP method to ensure strand specificity. RNA samples, namely, four samples from HEK293T cell lines under two conditions (control: wild‐type; case: heterogenous) with two biological replicates per condition, were obtained in FASTQ format. For each FASTQ file, quality checks were conducted using FastQC.^[^ ^59^ ^]^ Contaminating data, such as low‐quality reads, adaptor sequences, and poor‐quality bases, were removed with Trimmomatic software.^[^ ^60^ ^]^ Trimmed reads were mapped to the human reference genome (GRCh38) using STAR^[^ ^49^ ^]^ and then sorted by SAMtools.^[^ ^61^ ^]^

### Statistical Analysis

All results are presented as the mean ± 95% confidence interval. Statistical analysis was performed using R Studio (version 4.1.0). Differences between the two groups were analyzed using unpaired two‐tailed Student's t‐tests or Wilcoxon rank‐sum tests. For multiple comparisons, one‐way analysis of variance (ANOVA) with post hoc Tukey HSD tests were used. *P*‐values < 0.05 were considered statistically significant. *P < 0.05, **P < 0.01, ***P < 0.001, and ****P < 0.0001.

### eRNA‐QTL Atlas for Exploration and Analysis of Genetic Effects on eRNA

To enhance accessibility to eRNA‐QTL information, an intuitive and user‐friendly database (eRNA‐QTL atlas, http://bioinfo.szbl.ac.cn/eRNA‐QTL‐atlas/) was developed. The eRNA‐QTL search and query functionality was implemented within the robust database infrastructure, enabling users to conduct searches across 49 distinct human tissues. This advanced capability allows users to thoroughly explore the functional specific characteristics of eRNA‐QTLs by eRNA IDs (e.g., ENSR00000069687), thereby identifying associations with eRNA‐QTLs of particular interest (Figure S8A, Supporting Information). Furthermore, direct retrieval of eRNA‐QTLs is also included, which provides researchers with a sophisticated tool for their investigations. Then a genome browser was employed to visualize and explore the eRNA‐QTL genomic landscape surrounding target eRNAs by their genome positions or adjacent genes of target eRNAs. For example, eRNA‐QTLs in a specific tissue or all tissues for ENSR00000253886 can be shown by searching for its adjacent gene *TBX15*, which is positioned upstream of the adjacent region of ENSR00000253886 (Figure S8B, Supporting Information). These browsers provide an intuitive interface for navigating genomic regions and visualizing annotations, including gene structures, GWAS catalog risk SNPs,^[^ ^62^ ^]^ and eRNA‐QTLs among all 49 human tissues. By integrating the retrieved eRNA‐QTL data and genome browser visualizations, a comprehensive profile of the target eRNAs was constructed, shedding light on their potential function. In addition to these features, the ‘GWAS‐eRNA‐QTLs Colocalization Event Visualization’ section was offered by R package LocusComparer.^[^ ^63^ ^]^ This feature helps researchers conveniently visualize colocalization events between GWAS data and eRNA‐QTLs. An illustrative instance of the colocalization plot depicts the region surrounding enhancer ENSR00000089936, along with GWAS and eRNA‐QTLs *P*‐values, specifically in nerve tibial tissue (Figure S8C, Supporting Information). Furthermore, a compilation of eRNA‐QTLs associated with GWAS allowed users to delve deeper into the underlying mechanisms of eRNA‐QTLs in association with human traits and diseases. This compilation included full information on 1625 traits/diseases from the GWAS catalog across all 49 tissues (Figure S8D, Supporting Information). As an example, an instance of eRNA‐QTLs associated with the trait of telomere length in adipose subcutaneous tissue was presented. Finally, the download page allowed users to download all eRNA‐QTL results across 49 human tissues for further custom analysis (Figure S8E, Supporting Information). A comprehensive help page provides guidance for the effective utilization of these functionalities with a series of detailed, step‐by‐step instructions (Figure S8F, Supporting Information). Altogether, using the eRNA‐QTL atlas, researchers and investigators can efficiently access and analyze eRNA‐QTL data, empowering them to make informed decisions and gain valuable insights into the regulatory mechanisms underlying their studies related to eRNAs. Thus, the platform provides a powerful and scientifically rigorous experience for the exploration of eRNA‐QTL information.

### Data Availability

The data presented in this study can be freely accessed, queried, visualized, and downloaded through the dedicated eRNA‐QTL website portal at https://bioinfo.szbl.ac.cn/eRNA‐QTL‐atlas/. Sequencing data are deposited in the Gene Expression Omnibus (GEO) database (accession number: GSE242322) (token qfcjwyqmbjuptkf). Raw whole transcriptome and genome sequencing data from the Genotype‐Tissue Expression (GTEx) project are available via the database of Genotypes and Phenotypes (dbGaP) under the accession number: phs000424.v8.p223. All processed GTEx data are available via the GTEx portal((http://gtexportal.org/). GWAS summary statistics are from NHGRI‐EBI GWAS catalog (https://www.ebi.ac.uk/gwas/), UK Biobank GWAS (http://www.nealelab.is/uk‐biobank/), Finn Gen (https://www.finngen.fi/en), and JENGER (http://jenger.riken.jp). The details, including accession numbers, of GWAS summary statistics used in this study, are listed in Table S3. 1000 Genomes Project Reference and the regression weights used in LDSC analysis can be obtained at https://data.broadinstitute.org/alkesgroup/LDSCORE. All significant cancer susceptibility eRNAs identified by eRNA‐TWAS are provided in Table S6. The expression TWAS models for GTEx v8 are publicly available at PredictDB (https://predictdb.org/).

## Conflict of Interest

The authors declare no conflict of interest.

## Author Contributions

W.C., Z.W., and Y.W. contributed equally to this study. The study was conceptualized by L.L. Methodology was developed by W.Y.C. and L.L. Investigation was carried out by W.Y.C., Z.Y.W., Y.N.W., J.X.L., H.C., X.X.M., and X.D.Z. Visualization was done by W.Y.C., J.X.L., X.L., Y.N.W., Y.M.Q., and X.L.M. Funding acquisition was handled by L.L. Supervision was provided by L.L. and Y.B.Q. The original draft was written by W.Y.C., while L.L. and Y.B.Q. contributed to the writing and editing of the review.

## Supporting information



Supporting Information

Supplemental Table 1

## Data Availability

The data that support the findings of this study are openly available in http://bioinfo.szbl.ac.cn/eRNA‐QTL‐atlas. Sequencing data are deposited in the Gene Expression Ominbus database with the accession number GSE242322.
