# Supplementary material for: Enhancer RNA Transcriptome‐Wide Association Study Reveals a Distinctive Class of Pan‐Cancer Susceptibility eRNAs
Source: Adv Sci (Weinh). 2025 Feb 14;12(13):2411974. doi: 10.1002/advs.202411974 (PMC11967800; doi:10.1002/advs.202411974)
Supplement: Supplementary file 1 — Supporting Information [file ADVS-12-2411974-s002.docx]

Supplementary Materials for

**Enhancer RNA transcriptome-wide association study reveals a distinctive class of pan-cancer susceptibility eRNAs**

Wenyan Chen *et al.*

*Corresponding author. Email: Lei Li, [lei.li@szbl.ac.cn](mailto:lei.li@szbl.ac.cn); or Yunbo Qiao (ybqiao@shsmu.edu.cn)

**This PDF file includes:**

Figs. S1 to S8

**
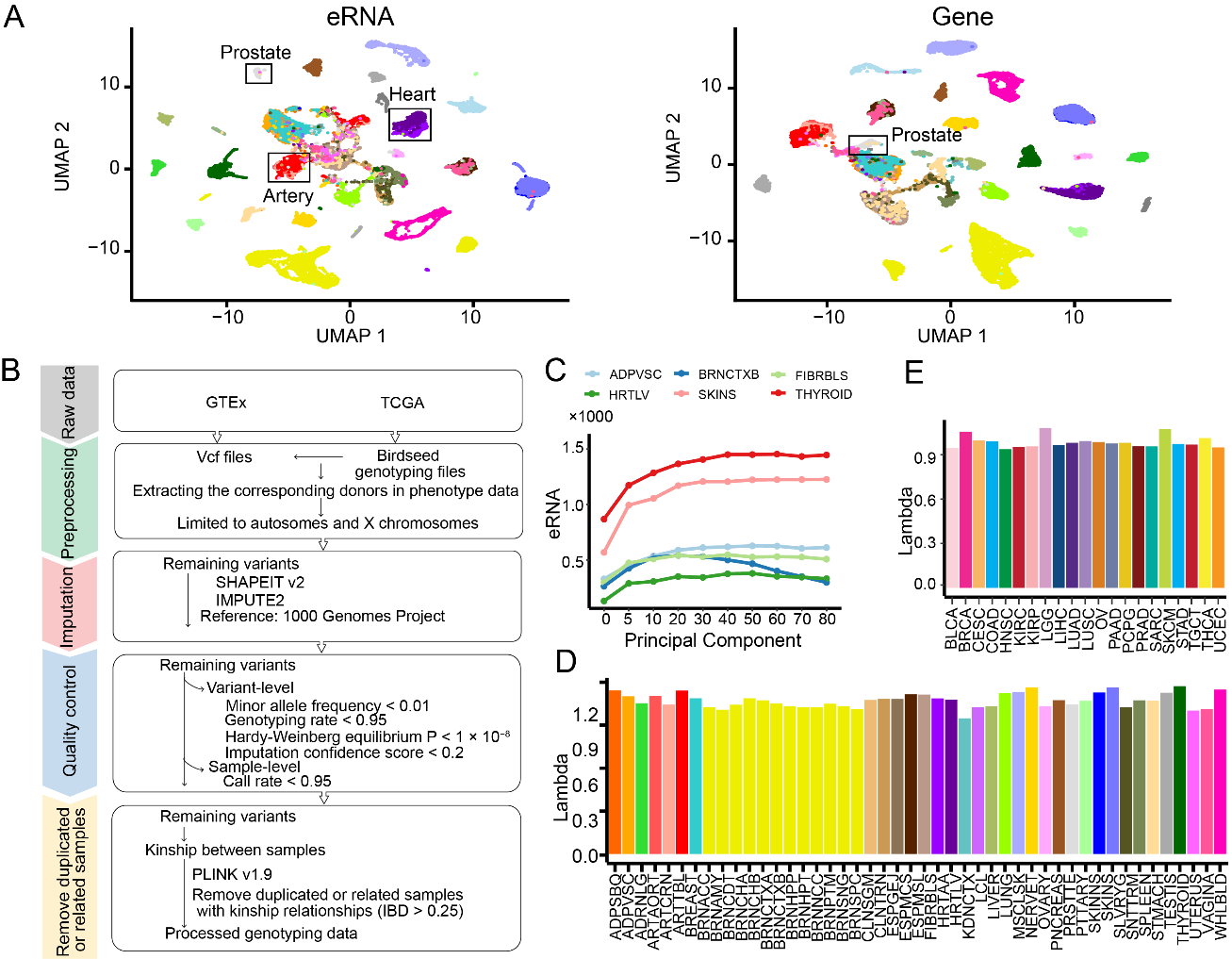
**

Fig. S1. Analysis of eRNA Expression and eRNA-QTL Mapping. (A) UMAP visualization of eRNA and gene expression stratified by tissue type. The plot depicts the clustering patterns of eRNAs and genes based on their expression profiles across 17,265 samples from various normal tissues within the GTEx project. Each dot's color and shape correspond to the tissue of origin as catalogued in the GTEx dataset, providing a comprehensive view of tissue-specific expression patterns. The color of each dot refers to the tissue recorded in the GTEx dataset (Table S1). (B) Genotyping data processing pipeline for GTEx and TCGA dataset. Our data has undergone thorough imputation, stringent quality control, and the removal of duplicated or related samples based on kinship relationships. (C) Significant eRNAs discovered across different phenotype PCs (FDR<0.05). The x-axis represents the number of phenotype PCs included in the eRNA-QTL mapping, while the y-axis corresponds to the number of significant eRNAs detected. (D) Assessment of genomic inflation factors for eRNA-QTLs across a range of normal tissues in the GTEx project. (E) Genomic inflation factors for eRNA-QTLs across diverse cancer types within TCGA.


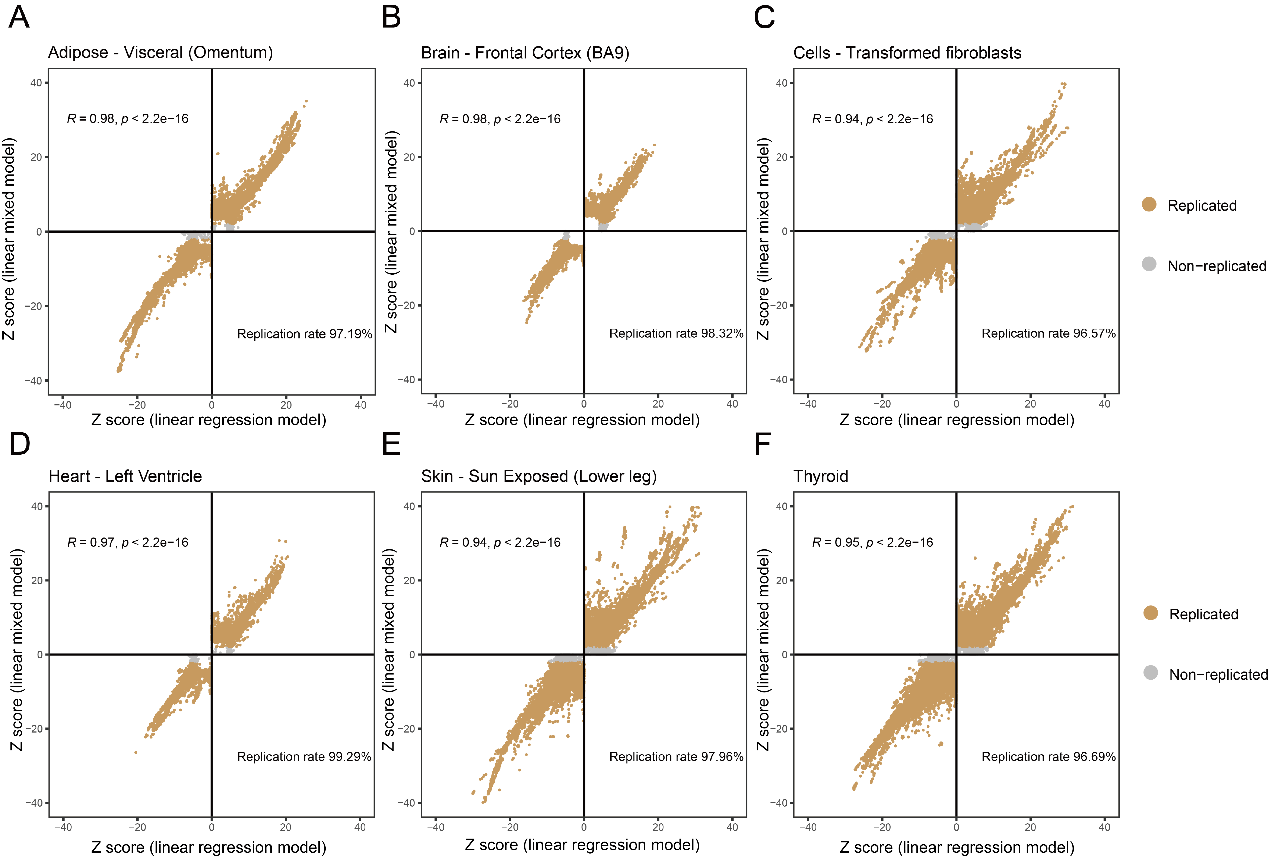


Fig. S2. **High concordance between the linear mixed model and the linear regression model in eRNA-QTL mapping across six representative tissues.** This figure shows the z-scores of eRNA-QTLs identified using both models across six tissues, with the x-axis representing the linear regression model and the y-axis representing the linear mixed model. (A) Adipose - Visceral (Omentum): The z-scores of eRNA-QTLs identified in adipose visceral (omentum) tissue using both models are shown, with a correlation coefficient of 0.98. (B) Brain - Frontal Cortex (BA9): The z-scores of eRNA-QTLs in the frontal cortex (BA9) of the brain are depicted, also exhibiting a correlation coefficient of 0.98. (C) Cells - Transformed Fibroblasts: The z-scores of eRNA-QTLs in transformed fibroblasts are presented, with a correlation coefficient of 0.94. (D) Heart - Left Ventricle: The z-scores of eRNA-QTLs in the left ventricle of the heart are displayed, showing a correlation coefficient of 0.97. (E) Skin - Sun Exposed (Lower Leg): The z-scores of eRNA-QTLs in sun-exposed skin (lower leg) are illustrated, with a correlation coefficient of 0.94. (F) Thyroid: The z-scores of eRNA-QTLs in thyroid tissue are shown, with a correlation coefficient of 0.95.


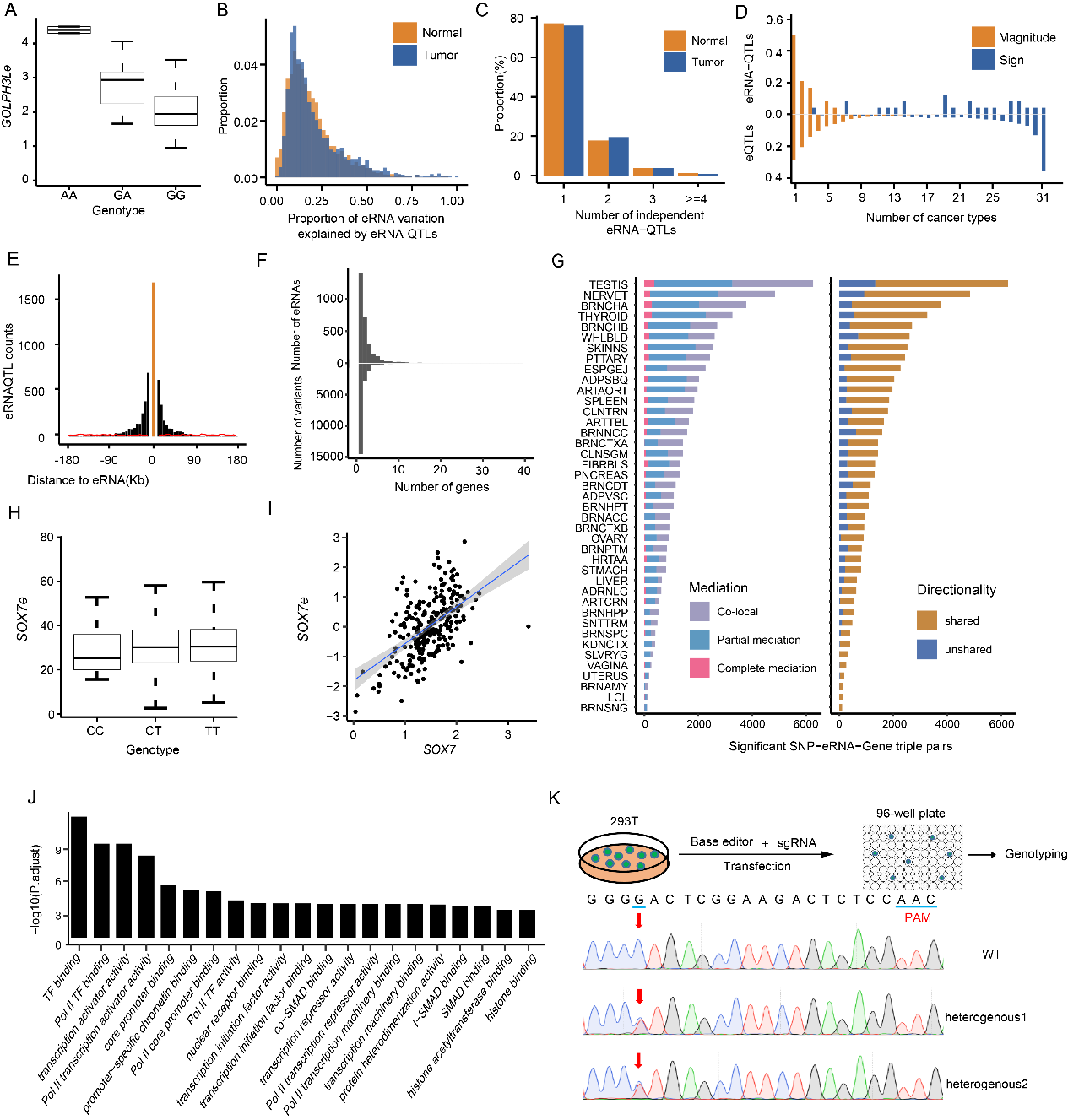


Fig. S3. **Functional Annotation of the eRNA-QTL**. (**A**) The example of SNP rs72700813 is closely related to the *GOLPH3L* eRNA (*GOLPH3Le*) in the brain cortex. Each dot in the box plot represents the expression (RPM) of *GOLPH3Le* in a particular individual (n = 184). Horizontal lines indicate the median values, and The boxes span the interquartile range between the 25th and 75th percentiles. The whiskers extend to 1.5× interquartile range (IQR). (B) Average fraction of eRNA variations that could be explained by eRNA-QTLs. The y-axis represents the proportion of eRNAs across all human normal (GTEx) tissues and tumor (TCGA) types studied. (**C**) Proportion of independent eRNA-QTLs across all human normal (GTEx) tissues and tumor (TCGA) types. (**D**) Proportion of tissues sharing lead eRNA-QTLs/eQTLs across all tumor tissues. (**E**) Locations of the lead eRNA-QTLs for eRNAs. The x-axis represents a candidate region divided into multiple bins, as described. The y-axis indicates the count of lead eRNA-QTLs within each bin, where each lead eRNA-QTL corresponds to a specific eRNA and represents the most significant eRNA-QTL. For eRNA-QTLs that fall outside eRNAs, they are assigned to bins based on their physical distance from the eRNAs. To provide a comparison, the red line represents randomly selected positions within a ±1-Mb window surrounding each eRNA. The yellow bar in the figure represents the eRNA region. (**F**) The number of variants, eRNAs and genes tested as candidate X-M-Y triplets. (**G**) The number of significant SNP-eRNA-Gene triple pairs in each tissue. Colors indicated mediation model and directionality. (**H**) The example of SNP rs17776622 that contained the YY1-binding site is closely related to the expression of *SOX7* eRNA (*SOX7e*). (I) The expression of *SOX7e* is tightly associated with *SOX7*. (J) Biological process for TFs whose eRNA-QTL were enriched. (K) Schematic diagram of the establishment of the monoclonal cell line with expected eRNA point mutations. The eRNA-mutant bulk cells were diluted and seeded onto a 96-well plate for culture. Single-cell clones were genotyped by Sanger sequencing after culture for 12 days. The site of rs6703982 was edited by BE4max-NG.


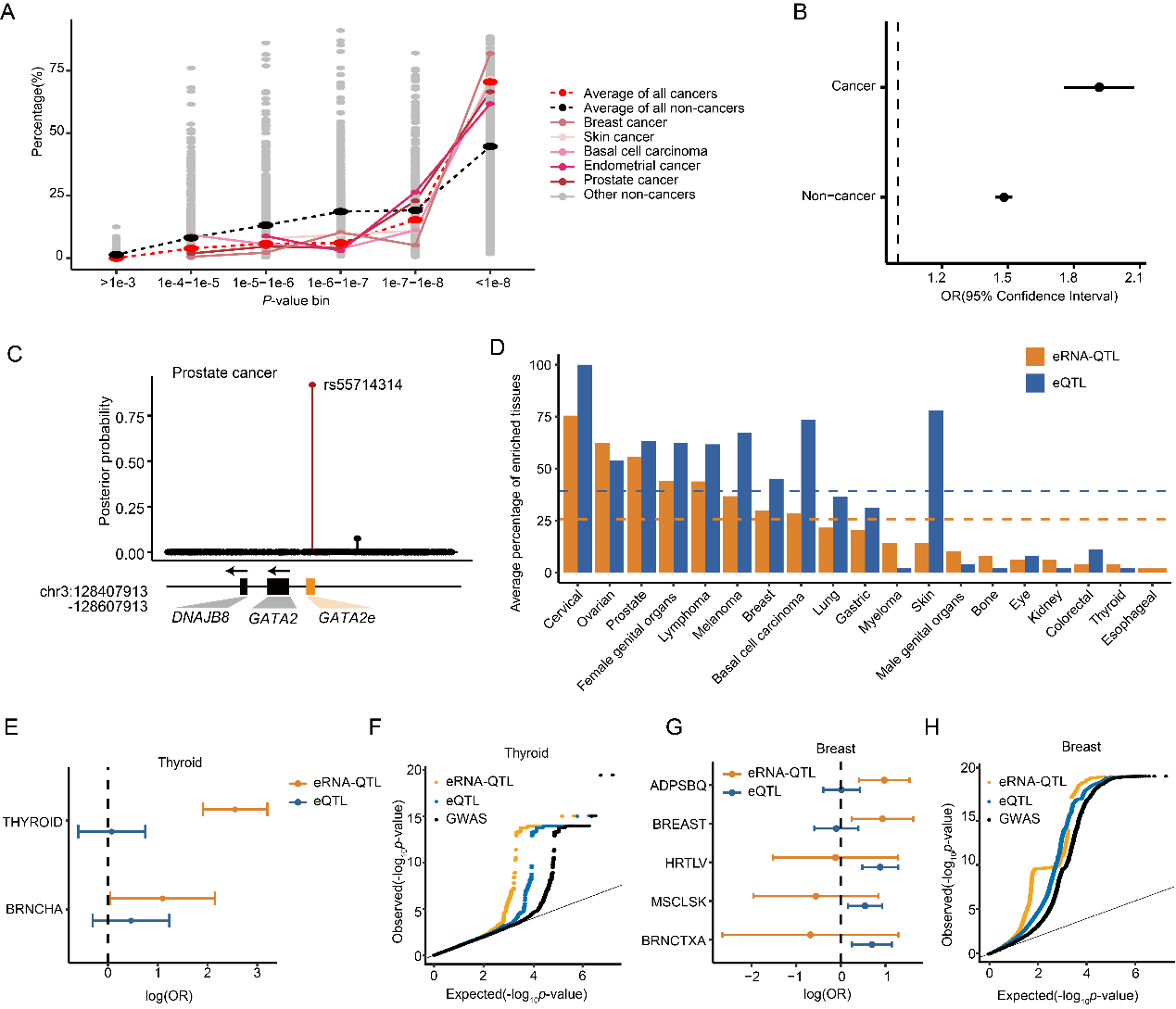


Fig. S4. **Functional Annotation of the eRNA-QTL**. (A) Fraction of GWAS variants above a given *P*-value threshold that overlap with an eRNA. The black dashed line represents the average fraction of all non-cancer GWAS variants above a given *P*-value threshold that overlap with eRNAs. The red dashed line represents the average fraction of cancer GWAS variants above a given *P*-value threshold that overlap with eRNAs. The red solid line indicates the fraction of cancer GWAS variants above a given *P*-value threshold that overlap with eRNAs. The gray dots represent the fraction of non-cancer GWAS variants above a given *P*-value threshold that overlap with eRNAs. (B) Cancer causal variants were more significantly enriched in eRNA transcribing regions than non-cancer causal variants. To investigate the genomic distributions of disease-associated fine-mapped variants (95% credible sets), we conducted an analysis of these variants within eRNA transcribing regions across various disease domains sourced from CAUSALdb. (C) The highest PP of a causal variant for prostate cancer was observed in eRNA regions. The *GATA2* eRNA (*GATA2e*) region contained eight causal SNPs associated with prostate cancer. Notably, rs55714314 (red color), an SNP located within the *GATA2e* region, exhibited the highest PP with prostate cancer. (D) Average percentage of enriched tissues across all cancer types. The x-axis represents various cancer types, while the y-axis indicates the number of tissue-trait pairs in which QTLs are enriched for each corresponding cancer type. The “tissue-trait pair” indicates the pairs of 49 tissues and 57 GWAS traits. The yellow dashed line represents the average number of tissue-trait pairs enriched with eRNA-QTLs across different cancer types. The blue dashed line indicates the average number of tissue-trait pairs enriched with eQTLs across different cancer types. (E) Tissues with the enrichment of at least one type of QTL for thyroid cancer. Estimated lower- and upper-bound 95% confidence intervals for enrichment values are also shown. The x-axis represents the enrichment level of QTLs in the corresponding cancer types, indicated by the log-transformed odds ratio (OR). The y-axis lists the tissues in which QTLs are enriched within each cancer type. The black dashed line denotes a log(OR) of 0, where values below 0 indicate no enrichment, and values above 0 indicate enrichment. Yellow and blue colors represent the enrichment patterns of eRNA-QTLs and eQTLs, respectively, across different tissue-trait pairs. Only tissue-trait pairs with at least one type of QTL enrichment are displayed. (F) Example quantile-quantile plot (QQ plot) showing the nominal *P*-values of thyroid cancer GWAS SNPs, which were primarily annotated by eRNA-QTLs (yellow) and eQTLs (blue). Each dot represents a GWAS SNP. All GWAS nominal *P*-values are also shown as controls (black). the X-axis represents the theoretical distribution of p-values for QTLs in GWAS, while the Y-axis represents the observed distribution of p-values for QTLs in GWAS. (G) Tissues with the enrichment of at least one type of QTL for breast cancer. (H) Example quantile-quantile plot (QQ plot) showing the nominal *P*-values of breast cancer GWAS SNPs, which were primarily annotated by eRNA-QTLs (yellow) and eQTLs (blue). Each dot represents a GWAS SNP. All GWAS nominal *P*-values are also shown as controls (black).


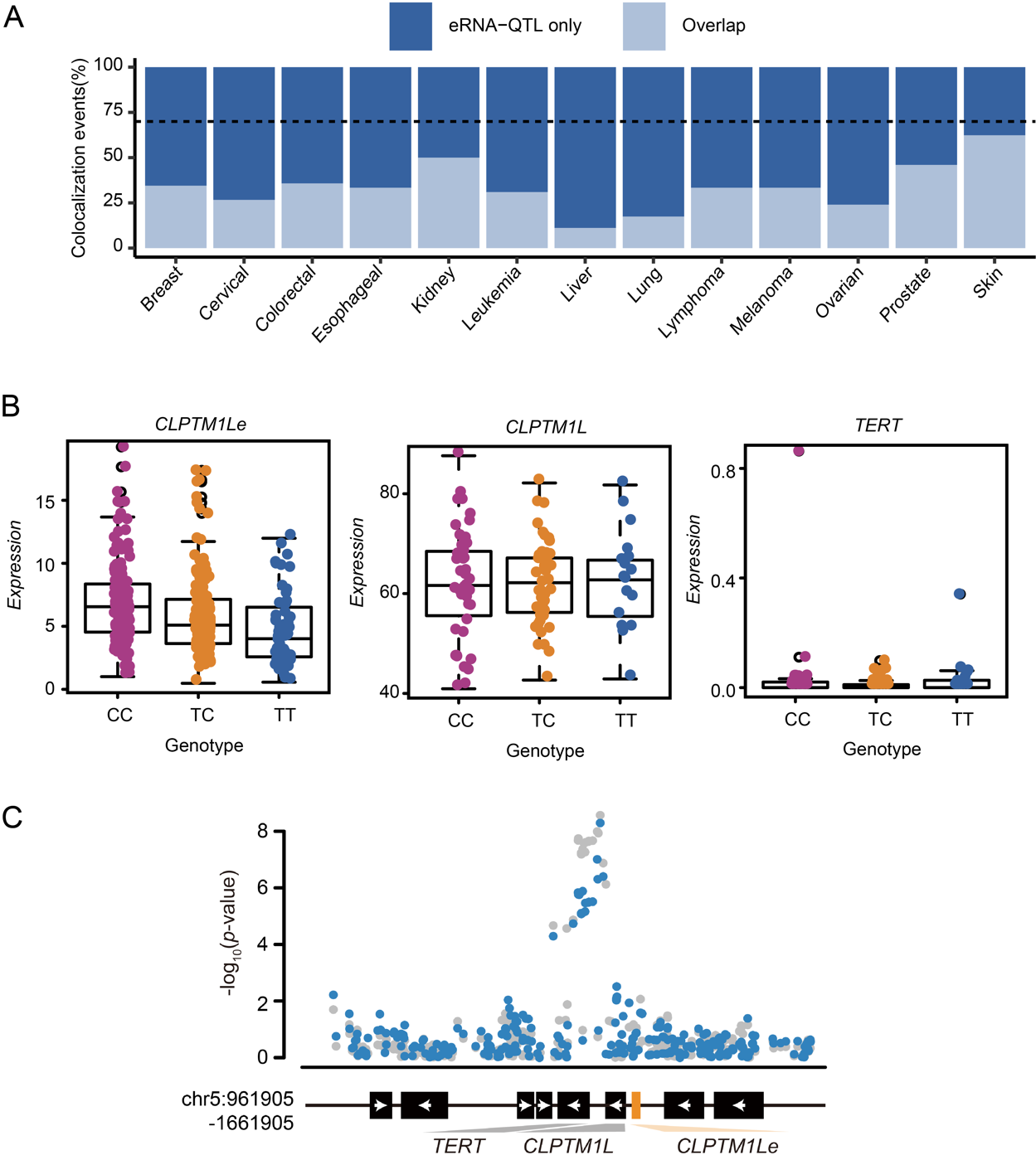


Fig. S5. Characterizing cancer risk loci and gene expression relationships. (**A**) The proportion of cancer risk loci that are co-localized with both eRNA-QTLs and eQTLs among those loci that are co-localized with eRNA-QTLs. Darker shades indicate that cancer risk loci are exclusively co-localized with eRNA-QTLs and not with eQTLs. Lighter shades signify that cancer risk loci are co-localized with both eRNA-QTLs and eQTLs. (**B**) The boxplot illustrates the correlation between the different genotypes of the eRNA-QTL rs6866783 and the expression of *CLPTM1Le*, *CLPTM1L*, and *TERT*. The x-axis represents the three distinct genotypes of rs6866783, while the y-axis values correspond to the expression levels of *CLPTM1Le* normalized by RPM, and the expression levels of *CLPTM1L* and *TERT* normalized by TPM. (**C**) Manhattan plot depicting cervical cancer GWAS signals before and after conditioning on *TERT* expression.


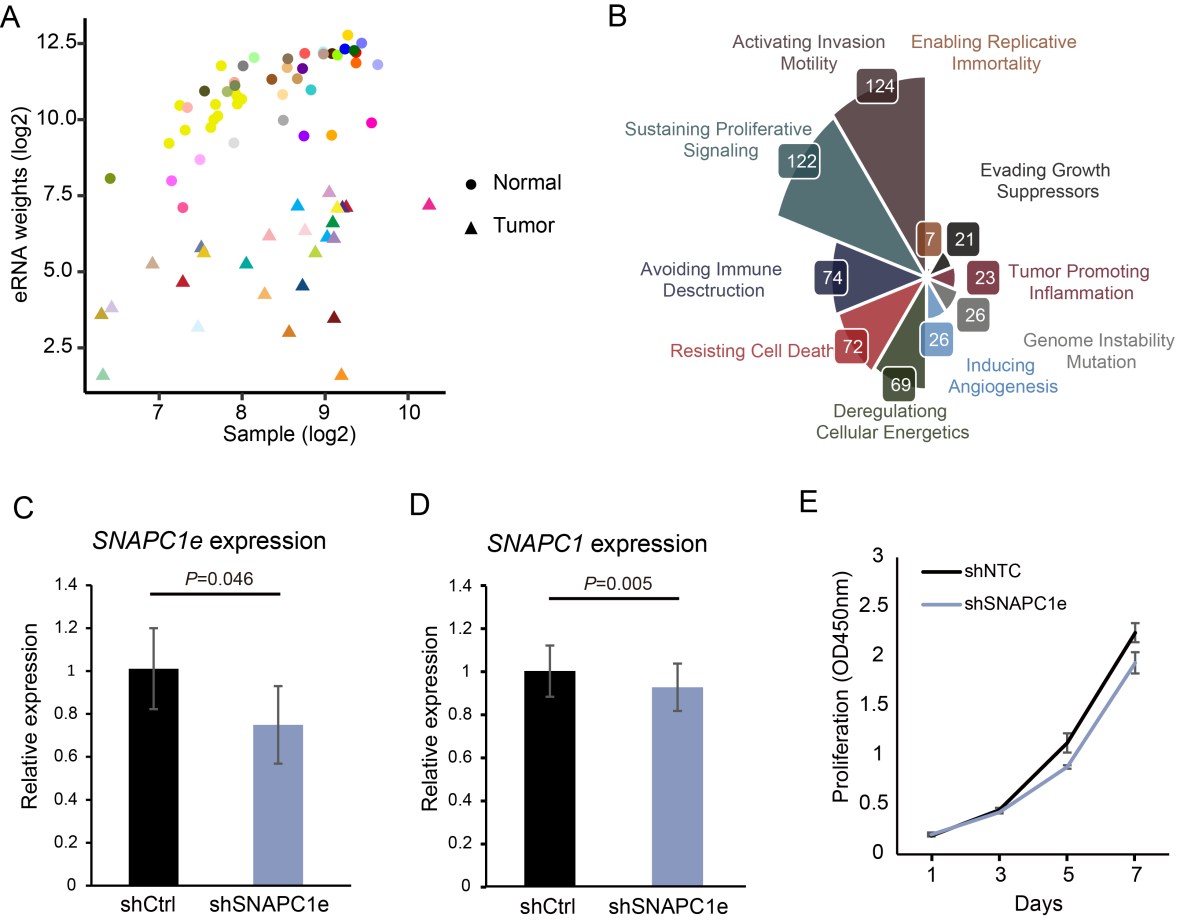


Fig. S6. The biological and clinical features of cancer susceptibility eRNA-link genes. (A) The number of eRNA-TWAS prediction models and the corresponding sample sizes of the reference panels are presented. Both the number of eRNA-TWAS prediction models and the sample sizes of the reference panels are transformed using a log2 scale. Circles represent normal tissues, while triangles represent tumor tissues. The color coding is detailed in Table S1. (B) 343 cancer susceptibility eRNA-link genes are known to contribute to the set of cancer hallmarks. (C-D) Quantitative reverse transcription (qRT)-PCR measuring indicated eRNA(C) and gene(D) expression upon *SNAPC1e* knockdown in PC3 cells. (n=3). (E) Cell proliferation of *SNAPC1e* knockdown cells was analyzed on days 1, 3, 5, and 7 (n = 3).


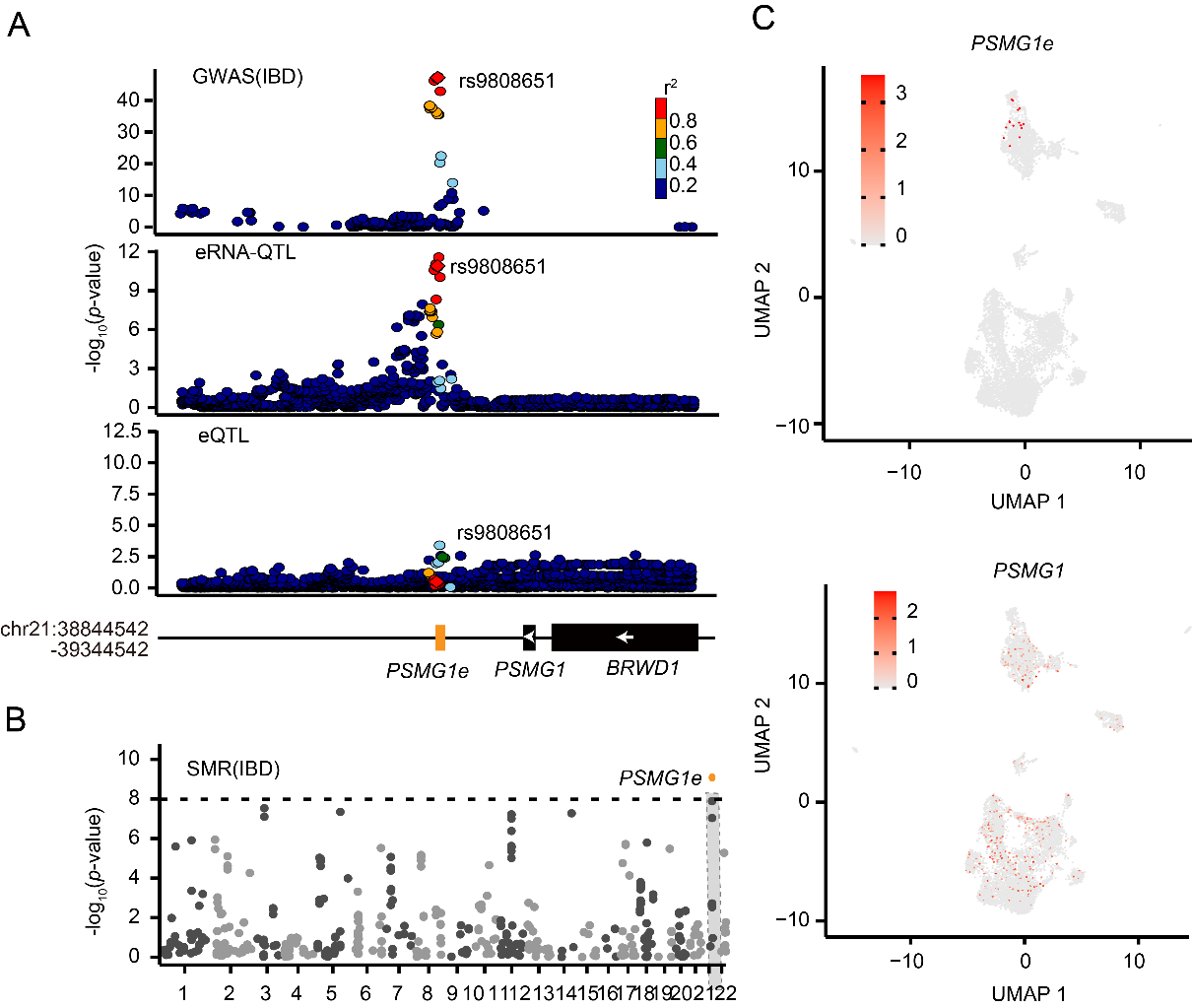


Fig. S7. **eRNA-QTLs contribute to a range of human phenotypes by regulating gene expression in a cell type-specific manner.** (**A**) Locus zoom plot of inflammatory bowel disease (IBD) GWAS signal; Shading of the points represents the linkage disequilibrium (r2, based on the 1000 Genomes Project Europeans; gray indicates unknown LD) between each SNP and the top SNP rs9808651, indicated by purple shading. Second panel, locusZoom plot showing the association with *PSMG1* eRNA (*PSMG1e*) expression. Bottom panel, locusZoom plot showing the association with *PSMG1* expression. (**B**) The Manhattan plot displays the SMR result of eRNAs relevant to IBD disesase. The dashes represent the significant threshold. (C) For the visualization at the single cell level, expression UMAP plots for *PSMG1e* and the *PSMG1* were generated. Colour bar indicates log2 normalized expression.


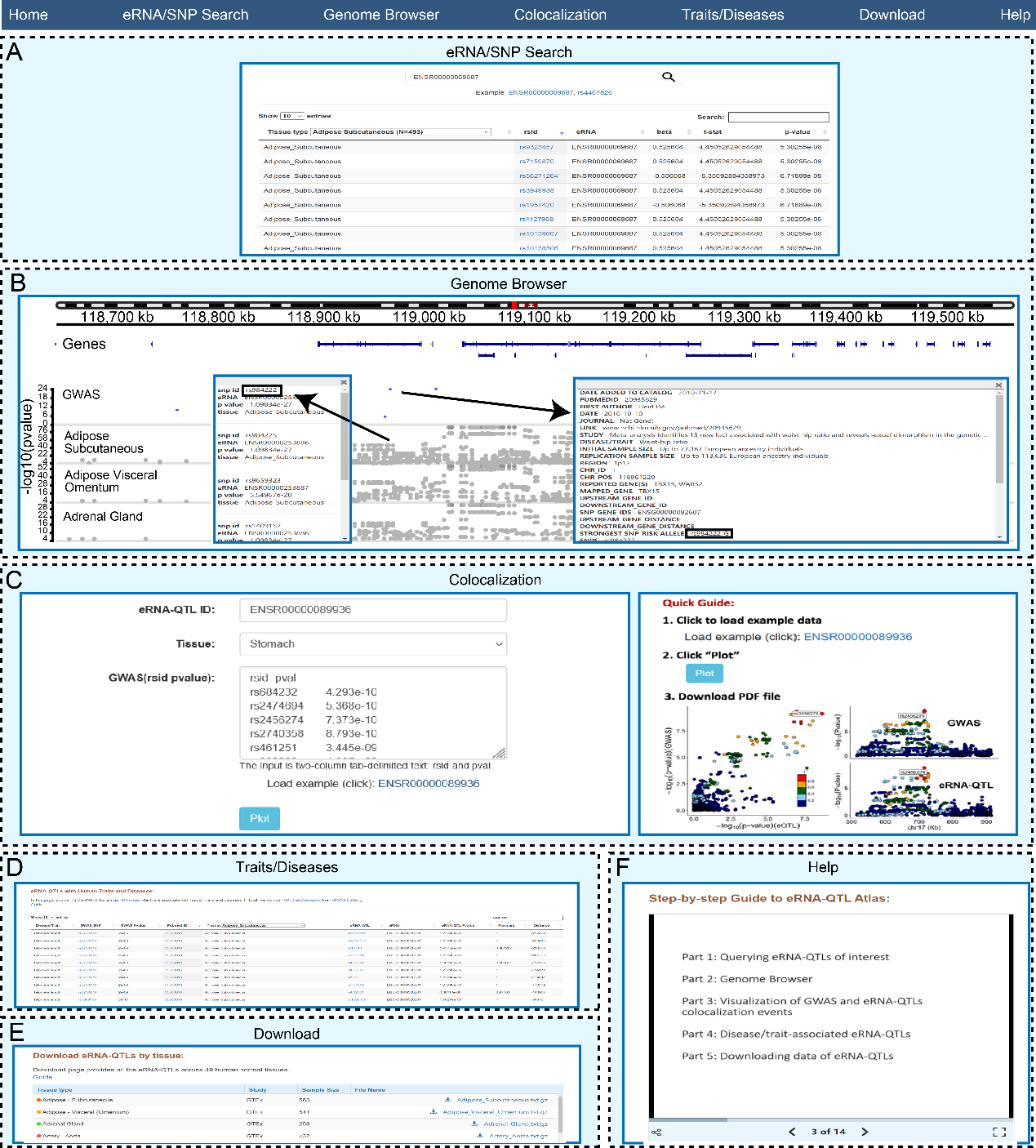


Fig. S8. **The web interface of eRNA-QTL atlas.** (**A**) eRNA-QTLs query interface and result visualization. (**B**) An example of the genome browser view shows the eRNA-QTLs in adipose subcutaneous tissue for ENSR00000253886 at the upstream of adjacent gene *TBX15*. (**C**) The interface of the ‘GWAS eRNA-QTLs Colocalization’ and an example of the LocusCompare plot at the region of enhancer ENSR00000089936 with GWAS *p-value* and eRNA-QTL *p-value* in stomach tissue. (**D**) The interface of the ‘Traits/Diseases’ and an example of the eRNA-QTLs with telomere length in adipose subcutaneous tissue. (**E**) Data download. (**F**) A detailed help page is provided to serve as a guide for users in understanding the usage of eRNA-QTL Atlas and interpreting the output data.
